# Effect of Elevated Temperature Thermal Aging/Exposure on Shear Response of FRP Composites: A Topical Review

**DOI:** 10.3390/polym18030354

**Published:** 2026-01-28

**Authors:** Rabina Acharya, Vistasp M. Karbhari

**Affiliations:** 1 Department of Civil Engineering, University of Texas Arlington, Arlington, TX 76006, USA; rxa5310@mavs.uta.edu; 2 Department of Mechanical and Aerospace Engineering, University of Texas Arlington, Arlington, TX 76006, USA

**Keywords:** fiber reinforced polymers (FRPs), thermal degradation, elevated temperature, thermal cycling, thermal spike, shear properties, interlaminar shear strength (ILSS), in-plane shear strength (IPSS), flexural strength, failure mechanisms

## Abstract

Fiber-reinforced polymer (FRP) composites are increasingly used in civil, marine, offshore, and energy infrastructure, where components routinely experience temperatures above ambient conditions. While the design of these components is largely driven by fiber-dominated characteristics, the deterioration of shear properties can lead to premature weakening and even failure. Thus, the performance and reliability of these systems depend intrinsically on the response of interlaminar shear characteristics, in-plane shear characteristics, and flexure-based shear characteristics to thermal loads ranging from uniform and monotonically increasing to cyclic and spike exposures. This paper presents a critical review of current knowledge of shear response in the presence of thermal exposure, with emphasis on temperature regimes that are below T_g_ in the vicinity of T_g_ and approaching T_d_. Results show that thermal exposures cause matrix softening and microcracking, interphase degradation, and thermally induced residual stress redistribution that significantly reduces shear-based performance. Cyclic and short-duration spike/flash exposures result in accelerated damage through thermal fatigue; steep thermal gradients, including through the thickness; and localized interfacial failure loading to the onset of delamination or interlayer separation. Aspects such as layup/ply orientation, fiber volume fraction, degree of cure, and the availability and permeation of oxygen through the thickness can have significant effects. The review identifies key contradictions and ambiguities, pinpoints and prioritizes areas of critically needed research, and emphasizes the need for the development of true mechanistic models capable of predicting changes in shear performance characteristics over a range of thermal loading regimes.

## 1. Introduction

Fiber-reinforced polymer (FRP) composites are increasingly used across a wide range of industries due to their exceptional mechanical performance, corrosion resistance, and lightweight nature [1]. In the range of applications, FRP composites are subjected to varying and harsh environmental conditions, including thermal exposure, moisture, ultraviolet (UV) radiation, and chemical exposure. The understanding of the material’s response to these environments over extended periods of time is crucial for assessing long-term durability and developing strategies to enhance the lifespan and reliability of the resulting components and systems [1].

Among these environmental conditions, thermal exposure plays a particularly critical role. Elevated temperatures, whether from prolonged exposure or intermittent spikes, can affect all constituents of the composite, including the interface, affecting the composite’s physical, chemical [2], and mechanical properties [3]. Under elevated temperature conditions, service environments vary widely depending on the application, including bridge decks (49 °C) [4], subsea pipelines (55 °C) [5], or in more demanding sectors like aerospace for aircraft wings and control surfaces (≈150 °C) [6] and offshore oil and gas operation (150–232 °C) [7]. The ranges mentioned here refer to operational, or localized, thermal exposure conditions to provide context and do not imply fire exposure, combustion, or post-fire performance assessment. In such environments, degradation typically progresses gradually, involving mechanisms such as matrix oxidation, microcracking, and fiber–matrix debonding [3]. These changes compromise the material’s ability to transfer loads, leading to gradual performance deterioration [8]. In contrast, exposure to fire introduces a more aggressive and rapid chemical breakdown [9], where failure is primarily driven by thermal decomposition of the matrix and eventually the fibers themselves [10]. Fire exposure includes all the degradation mechanisms seen under elevated temperature but at a much faster rate, often accompanied by pyrolysis of the resin into volatile gases, smoke, and char, ultimately resulting in catastrophic failure [11,12]. The pre-charring phase of fire exposure is particularly critical, as the onset of polymer matrix decomposition, accompanied by delamination and matrix cracking, leads to a rapid loss of mechanical strength [9]. For clarity, it is emphasized that this review focuses on elevated temperature exposure and thermal aging scenarios relevant to service and near-service conditions, including sustained, cycling, and short-duration thermal excursions below decomposition and combustion thresholds. Fire exposure, post-fire residual performance, matrix pyrolysis, and combustion-driven systems-level collapse, while all important, represent distinct phenomena governed by different physical and chemical processes and are beyond the scope of the present review.

A critical determinant of a composite’s thermal behavior is the nature of the temperature exposure profile. In FRP composites, overall performance can vary significantly depending on whether the material is subjected to sustained (continuous or isothermal) heating or to non-uniform conditions such as thermal cycles or even temperature spikes. Sustained thermal exposure often results in gradual and irreversible matrix degradation, accompanied by scission of polymer chains and deterioration of the fiber–matrix interface [13]. In contrast, cyclic loading, which is defined as repeated cycles of heating and cooling, or thermal spikes, defined as periods of sudden temperature rise, induce fluctuating internal stresses that can initiate and propagate microcracks, ultimately leading to thermal fatigue and structural failure [14]. Temperature fluctuations can cause cyclic stresses due to a mismatch of thermal expansion coefficients of the constituents and can further result in differential stresses generated due to the stacking sequence. Both can result in interfacial cracking, which can be more detrimental than the effects due to isothermal heating at elevated temperatures.

**Figure 1:**
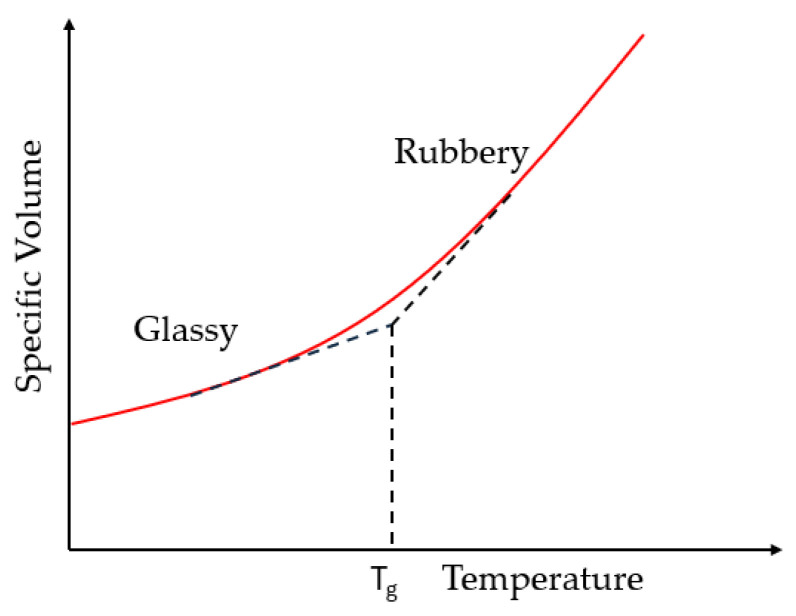
Specific volume change with temperature.

This overall response or change in the FRP composite under thermal exposure can be linked to two key temperature levels, the glass transition temperature (T_g_) and the decomposition temperature (T_d_). Glass transition temperature is the temperature at which the material changes its response from glassy and stiff to ductile and flexible due to softening of the matrix [3,15], with molecular chains gaining the ability to move freely due to an increase in free volume [16] and specific volume [17] as shown schematically in Figure 1.

**Figure 2:**
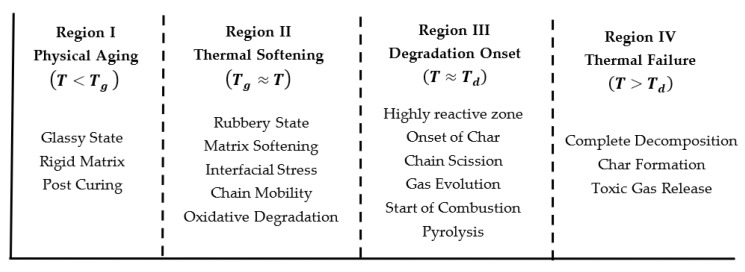
Temperature-dependent regimes governing shear-related mechanical response of FRP composites under thermal exposure.

In ambient-cured systems typically used in civil infrastructure rehabilitation, when exposed to sustained thermal load, the matrix can begin to soften at temperatures ranging from 60 °C to 82 °C [18]. Furthermore, the decomposition temperature, which marks the onset of chemical and irreversible degradation of the FRP composite, ranges from 250–400 °C for resins, depending upon the resin chemistry [19,20] and use of elevated temperature cure processes; 950 [21]–1000 °C [18] for carbon fiber and 980 [22]–1500 °C [9] for glass fiber. This pronounced difference in degradation temperature between the matrix and fibers highlights the vulnerability of the matrix in FRP systems during thermal loading; that is, as the temperature rises, the matrix undergoes thermal softening and decomposition first, while the fibers remain largely unaffected until significantly higher temperatures are reached [22]. Thus, matrix-dominated characteristics are affected significantly earlier, and hence their behavior is highly critical for the overall service response. From this perspective, the decomposition temperature marks the upper limit beyond which degradation dominates, and shear-based mechanical characterization is no longer meaningful. The overall response relevant to shear behavior can be characterized by four regions bounded by the glass transition temperature and the decomposition temperature, as shown in Figure 2. It is emphasized that rather than being distinct regions, these can show significant variation and overlap based on the specifics of cure and fiber loading. In the present context, the decomposition temperature should be considered as a material-specific upper bound defining the limit of applicability of mechanical response models rather than as a regime of structural relevance. The mechanical behavior discussed herein is restricted to temperatures below T_d_, where structural integrity is retained, and shear-dominated response remains physically meaningful. It is emphasized that the schematic in Figure 2 is intended to illustrate temperature-dependent transitions and mechanical response rather than to represent fire or combustion scenarios. Regimes beyond T_g_ and approaching T_d_ are included to delineate the boundaries at which shear-based mechanical characterization loses physical relevance.

While tensile characteristics are most often used in design, these are intrinsically fiber-dominated and thus do not show early effects of degradation. However, properties dominated by resin response, particularly shear properties, can be of primary concern to overall structural integrity. These properties are highly sensitive to changes in both the matrix material and the fiber–matrix interface, both of which are significantly compromised by elevated temperatures [23]. It is thus surprising that shear-related characteristics have received considerably less research attention under thermal loading [24].

This paper is thus focused on reviewing the effect of both sustained and non-linear thermal load (elevated temperature only) prior to charring on shear-dominated properties of CFRP and GFRP through the consideration of interlaminar shear strength (ILSS), which measures the shear strength between adjacent plies; in-plane shear strength (IPSS), which measures the shear strength of a lamina within its own plane; and the modulus. Furthermore, the changes in flexural strength and flexural modulus under thermal loading will also be reviewed since the complex response is highly relevant in this context because flexural failure often originates in the shear-critical matrix and interface regions, where the stresses concentrate and compromise the integrity of the composite, leading to delamination and interlayer separation, serving as an indicator of the overall performance and durability of composite materials when subjected to thermal stress [3].

To provide a comprehensive assessment of how thermal exposure influences shear-dominant failure mechanisms, this review initiates with a discussion of the fundamental mechanisms of thermal loading (both sustained and cyclic/spike) on FRPs, covering the effects of thermal exposure, coefficients of thermal expansion, temperature profiles, and commonly used shear testing methods. The effects of temperature on mechanical properties are then discussed, followed by a brief review of analytical models, leading to an overall review of findings from the study of composite materials’ thermal behavior in shear-dominated properties, including commonalities, contradictions, and gaps, followed by a summary and conclusions.

## 2. Fundamental Mechanisms and Behavior

The behavior of FRP when subjected to elevated temperature can be divided into four different phases, depending on two key temperature levels (T_g_ and T_d_) [23,25] as shown in Figure 2. In these four phases, the FRP undergoes two types of aging: physical and chemical [26]. While the vicinity of T_g_ is examined in this review to elucidate degradation mechanisms and failure mode transitions, it is not representative of typical design conditions. We generally limit service temperatures to well below T_g_ to preserve key mechanical characteristics such as stiffness and interfacial integrity. Physical aging primarily occurs when the FRP composite is subjected to a temperature below T_g_, and the specimen only changes at the level of molecular conformation without changing the structural integrity or breaking chemical bonds of the molecules [27]. These changes result in the densification of polymers with a slowly reducing free volume toward a more thermodynamically stable state [28]. In contrast, when a specimen is subjected to temperatures that are near or above T_g_ for an extended period or is subjected to temperatures near or above T_d_ even for a short period, chemical aging can occur, leading to irreversible degradation [29] and a reduction in molecular weight [30]. This occurs due to the breakage of bonds with higher dissociation energy (i.e., covalent bonds), resulting in chain scission, oxidation, and depolymerization [9]. Since the stress generated between fiber and matrix due to the difference in coefficient of thermal expansion (CTE) during thermal aging can also significantly influence the strength of the FRP composite [31], it is important to briefly review this material characteristic.

The coefficient of thermal expansion (CTE), which governs the dimensional stability of a material in response to changes in temperature, is highly dependent on the constituent materials (fibers and matrix), their volume fractions and configuration, and the operating temperature [32]. In FRP composites, a significant thermal mismatch exists between the low-CTE fibers and the higher-CTE polymer matrix [33,34] (typical CTE values are given in Table 1), with materials like carbon fibers even displaying a negative CTE in the longitudinal direction and positive CTE in the transverse direction [33,35].

**Figure 3:**
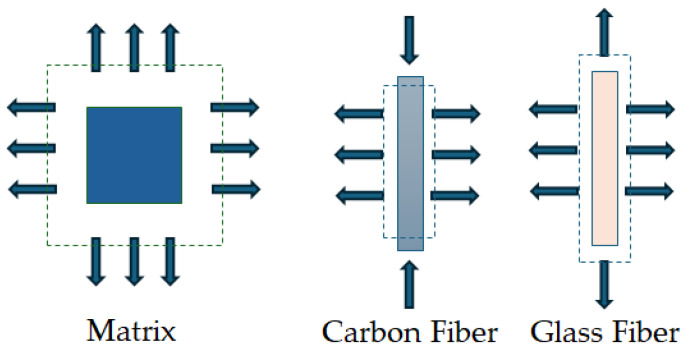
Schematic showing dimensional behavior of composite constituents with an increase in temperature (solid lines indicate original dimensions, new dimensions are shown by a dashed line, arrows indicate the changes in dimensions because of temperature).

As the temperature increases, the carbon fiber experiences contraction in the longitudinal direction and expansion in the transverse direction [33,35]. Conversely, glass fiber and the polymer matrix undergo expansion in both directions with an increase in temperature and a decrease as the temperature decreases, as shown schematically in Figure 3.

Due to the difference in CTE, the expansion/contraction of the fiber occurs at a comparatively lower rate than that of the matrix, leading to the development of differential thermal strains [33]. Cooling from the temperature of cure, or the exothermic temperature attained through the polymerization process, to room temperature results in the development of compressive stress in fibers and tensile stress in the matrix [38], with the fiber–matrix interphase experiencing tensile stress [39]. These internal residual stresses are most concentrated at the fiber–matrix interphase, where they can lead to the breakage of the relatively weak bond and the eventual initiation of matrix microcracking [32,33]. Even if residual stresses do not cause immediate damage, or can be controlled to some extent by lowering the cooling rates and allowing more time for stress relaxation in the glass transition region [40], they can still play a critical role over the structure’s lifetime. Under external loading, the combined effect of residual and applied stresses can trigger fiber–matrix debonding, matrix cracking, or delamination at interfaces between layers with different fiber orientations [41]. This phenomenon directly influences shear transfer across the interphase, ultimately affecting the material’s shear response. Since the CTE is influenced by several factors, including temperature, variations in the temperature profile alter the rate at which the CTE changes, thereby modifying both the rate and amplitude of residual thermal strains generated within the interphase [42], which ultimately affects the shear response of the composite material subject to thermal loading.

**Figure 4:**
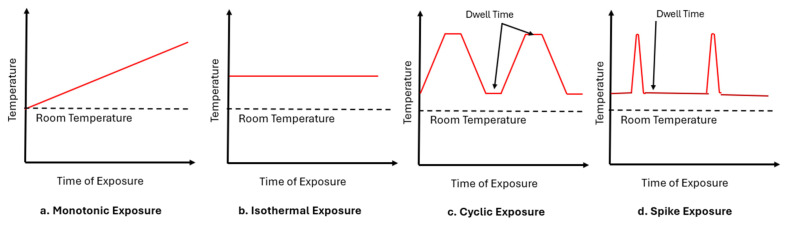
Schematic of types of temperature exposure profiles.

Thermal degradation mechanisms of FRPs are profoundly influenced by the nature of the temperature exposure, as shown schematically in Figure 4. Exposure conditions such as isothermal ageing generally result in property degradation, which is primarily time-dependent [13]. In contrast, a monotonic temperature increase represents a rate-dependent degradation that is a synergistic function of both time and temperature [3,43,44]. Non-uniform exposures, which more accurately simulate real-world service conditions, involve more complex degradation mechanisms. Thermal cycling leads to a gradual accumulation of damage through thermo-mechanical fatigue caused by the CTE mismatch between the polymer matrix and the reinforcing fibers, with the extent of dwell time being a critical variable. Conversely, thermal spikes induce immediate damage due to exposure to steep thermal gradients over a very short time period, which can cause catastrophic failure in a single event if a critical threshold is exceeded [45,46].

**Figure 5:**
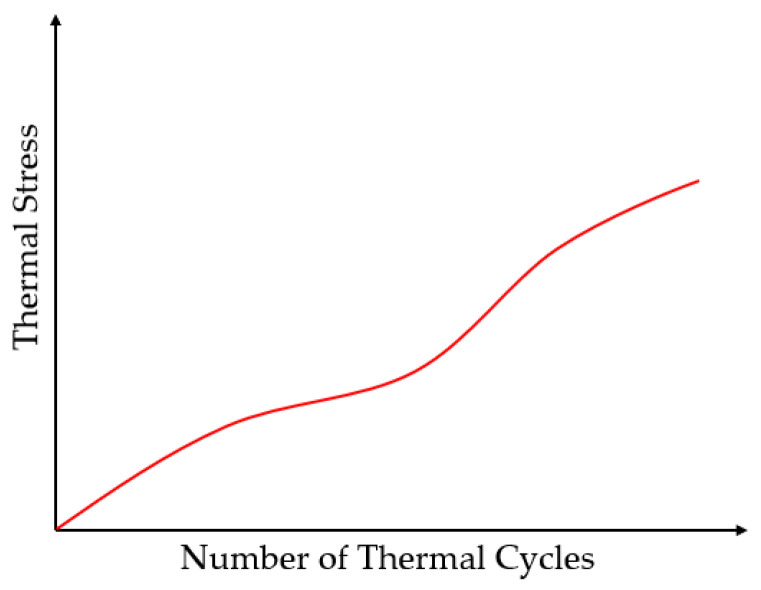
Increase in thermal stress with an increase in the number of thermal cycles.

As previously discussed, one of the causes of thermal degradation in composite materials is the significant thermal expansion mismatch between the fibers and the polymer matrix resulting from the difference in CTE. This mismatch creates a state of residual stress that is exacerbated by repeated thermal loading. Hancox [38] provided a theoretical framework explaining this phenomenon, deriving an expression that quantified the thermal stress generated by the difference in the CTEs between the fiber and matrix as

(1) $$\alpha_{L}≅-∆\alpha ∆TE_{H}$$

where α is the coefficient of thermal expansion, ∆T is the change in temperature, E is the modulus, and L and H refer to constituents with the lower and higher property characteristics, respectively. This framework shows that with every thermal cycle, additional stress is induced, as shown in Figure 5, which can lead to a range of damage, including transverse laminate cracking, debonding, and ply delamination. Research by Bailey et al. [47] and Jones et al. [48] further discuss the progressive deterioration of both longitudinal and transverse properties due to the accumulation of thermal strains and stresses in cross-ply laminates.

**Figure 6:**
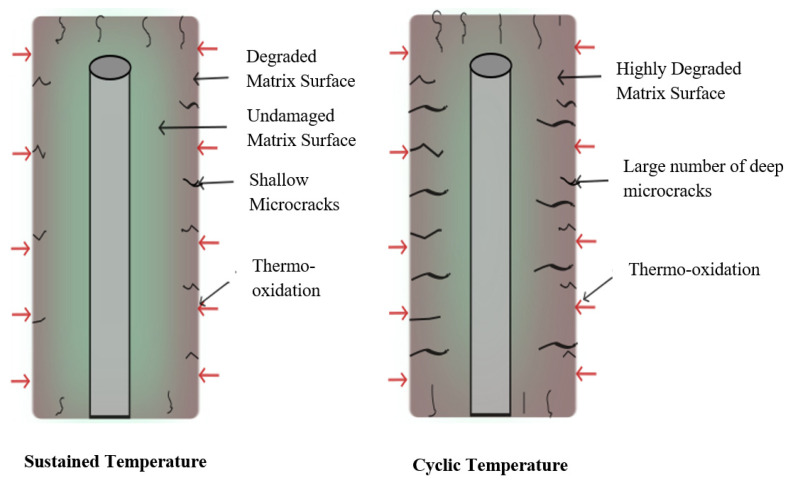
The effect of sustained and cyclic temperature on FRP composite.

While both isothermal aging and thermal cycling involve oxidation, damage mechanisms accruing from these loadings are distinct. Studies have shown that isothermal aging primarily degrades only the surface of the composite through gradual oxidation and matrix degradation [14], resulting from two competing phenomena: crosslinking and mass loss [49]. Thermal cycling in contrast couples this oxidation with a fatigue-like mechanism caused by cyclic stresses, which significantly accelerates the damage process, degrading both the surface and the core of the laminate [14]. This phenomenon is shown schematically in Figure 6.

The repeated cycles of heating and cooling reactivate molecular motion, enhancing oxygen diffusion at a microscopic level and triggering microcracks [50]. As a result, even when mass loss is equivalent in both cases of exposure, thermal cycling induces more pronounced matrix shrinkage, leading to earlier initiation and significantly greater accumulation and propagation of cracks compared to isothermal aging. This leads to more widespread damage throughout the material, from the surface to the core of the laminate [14,38]. Therefore, the extent and rate of microcrack propagation under thermal cycling or spiking are noted to be significantly higher than under monotonic or isothermal aging [51,52].

When FRP composites experience different temperature profiles, degradation mechanisms such as matrix softening, interfacial debonding, and microcracking can significantly impact shear-dominated properties, which often act as a limiting factor for overall use. Under conditions of thermal loading, failure mechanisms may shift, matrix failures can become dominant, interfaces may weaken, and damage can localize faster. To understand these changes, it is essential to first establish a baseline by understanding the standards used and what constitutes acceptable performance at room temperature. This will then enable the development of an understanding of the changes in failure mechanisms.

The inherent anisotropy of fiber-reinforced polymer matrix composites necessitates the use of a large set of tests to fully characterize material properties and to comply with design and structural requirements since no single test method is able to provide general failure information in all states of stress [53,54]. While numerous shear testing methods have been developed to evaluate the performance of composite materials, each method has its own set of advantages, limitations, complexities, and potential sources of inaccuracies [53]. An ideal shear test should yield reproducible results, should be easy to compute and economical to implement, and should successfully isolate the specific shear property of interest [55]. Therefore, understanding the differences between various test methods and their corresponding acceptable failure mechanisms is essential for accurately evaluating the performance of composite materials under specific loading conditions and for selecting the most suitable test method. Since thermal loading alters failure mechanisms and can compromise the validity of test methods, as will be discussed later in this paper as damaged regions may no longer be in composite action [56], it is important to assess different shear test methods and their acceptable failure modes. In general, these can be classified as related to interlaminar shear, in-plane shear, and flexural mechanisms, with test standards under each being listed below.

- *Interlaminar Shear* [57]: Short-Beam Shear Method (ASTM D2344/D2344M [58], ISO 14130 [59]), Double-notched Shear Method (ASTM D3846 [60]), Iosipescu Shear Method (ASTM D 5379/D5379M [61]);
- *In-Plane Shear* [55,62,63]: Plate Twist Method (ISO 15310), Punch Shear Method (ASTM D732 [64]), Short-Beam Shear Method (ASTM D2344/D2344M [58], ISO 14130 [59]), Iosipescu Shear Method (ASTM D 5379/D5379M [61]), Rail Shear Method (ASTM D7078/D7078M [65]), In-plane Shear Properties of Hoop Wound Polymer Matrix (ASTM D5448/D5448M-22 [66]), 10° off-axis tensile test [67], ±45° Tensile Test (ASTM D3518/D3518M [68]), slotted tensile specimen [69], cross-beam shear test [69], torsional shear of a thin-walled tube [70];
- *Flexure* [63]: Three-Point Flexural Test (ASTM D7264/D7264M [71], ISO 14125 [72]), Four-Point Flexural Test (ASTM D6272 [73], ASTM D7264/D7264M [71]).

**Figure 7:**
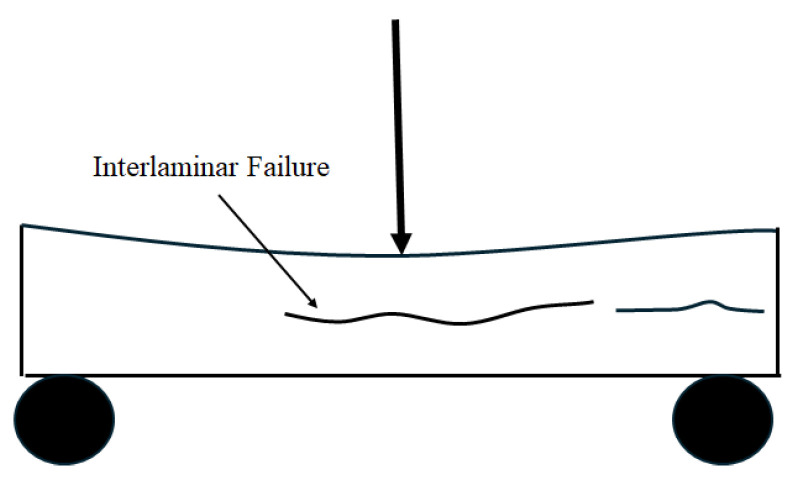
Acceptable failure mode during the short-beam shear strength test.

Due to the laminated structure, interlaminar stresses develop at the interface between adjacent plies/layers and can lead to delamination or interlayer separation, with the former being seen in laminated prepreg-type composites and the latter being reflected in wet layup-based composites. In both cases, stresses at the interlayer level can lead to unstable crack growth and overall catastrophic failure [74], with the threshold stress level referred to as the interlaminar shear strength (ILSS). Among various methods to measure ILSS, the short-beam shear (SBS) strength test is commonly used due to its simplicity, ease of specimen preparation, and suitability for evaluating interlaminar shear properties in laminated composites [75]. The main goal of this test is to induce a longitudinal crack perpendicular to the applied load and parallel to the fiber direction so that the shear strength between the matrix and fibers can be measured [76], as shown in Figure 7. This is achieved by minimizing the bending moments through the use of extremely short spans such that the through-thickness shear stress reaches its limiting value before normal stress does [77], and the specimen fails in the interlaminar region in the neutral plane [78]. Both ASTM D2344/D2344M-22 [58] and ISO 14130 [59] provide standards for the SBS test. The acceptable failure mode is shown schematically in Figure 7, with failures involving tension, compression, or plastic shear not being acceptable [59].

**Figure 8:**
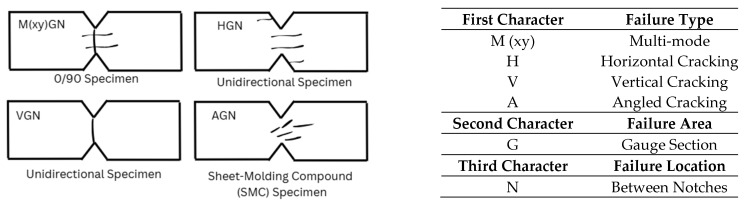
Acceptable failure modes during a V-notched/Iosipescu shear test [adapted from [60].

In-plane shear strength (IPSS) provides a measure of the composite laminate’s resistance to shear stress within the plane of the fibers. In-plane shear stress in composite materials arises within the laminae when forces act parallel to the plane of the material, causing layers or fibers within the composite to slide relative to each other. Several standard test methods exist for evaluating in-plane shear properties, with ASTM D5379 (V-notched/Iosipescu shear test) [61] and ASTM D3518 (±45° tension shear test) [68] being the most commonly used [79]. The Iosipescu shear test utilizes a V-notched specimen loaded in compression [80], whereas the ±45° shear test involves tensile loading [81]. The Iosipescu shear test is considered if a pure shear stress state in a uniform region is required, while the ±45° tensile test is preferred when a simple, economical test is required [63]. It should be noted that during a ±45° tensile test, normal stresses (tensile and compressive) are also present in the lamina, which can influence the results [81]. The failure mode that is reported for the ±45° tensile test is delamination fracture parallel to the fiber direction and interlayer separation in the bulk resin between the fabric layer [3]. Failure modes for the Iosipescu shear test are shown schematically in Figure 8 [61].

**Figure 9:**
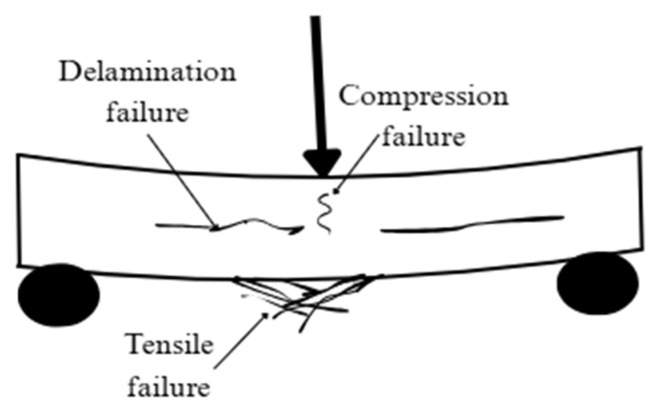
Acceptable failure modes during the flexural test.

While flexural tests determine the ability of FRP composites to resist bending loads [82], the composite laminates experience tensile stresses on the outermost fibers and compressive stresses on the inner fibers, while shear stresses develop in the mid-plane [83] making this a convenient test to assess shear response between layers. The three-point (ASTM D6272 [73] and ISO [72,84]) and four-point (ASTM D6272 [73]) bending tests are the most widely used methods for measuring flexural strength. The primary difference between the three-point and four-point flexure test is the occurrence of maximum bending moment and maximum flexural stress. In the three-point flexure test, vertical shear force and bending moment are concentrated around the loading nose, while in the four-point test, they are distributed throughout the specimen [85]. Thus, for cases where identifying shear failure is critical, three-point bending is employed, since it induces localized stress concentrations that make fracture initiation easier to detect. The acceptable failure mode is shown schematically in Figure 9 [72].

It is important to briefly point out the differences between the tests and mechanisms activated by thermal loading. Interlaminar shear tests show great sensitivity to matrix softening, microcracking, and fiber–matrix debonding, with ILSS decreasing rapidly due to the low resistance to crack propagation between layers. Aging above the glass transition temperature can accelerate plasticization, chain scission, and weaken the interfacial regions, resulting in the steepest decline in characteristics with increase in temperature among the three types of tests. In comparison, in-plane shear tests show performance degradation due to temperature-driven matrix softening and interlaminar matrix microdamage, both of which occur at a slower rate than the interlaminar mechanisms highlighted by the ILSS tests. Thermal aging close to, or above, T_g_ can effectively decrease the shear modulus, with matrix yielding leading to decreased shear stain and load transfer capacity. Flexural tests are influenced by both inter- and intra-laminar shear, leading to a more complex response, with higher temperatures leading to compression side micro-buckling instabilities that may exacerbate shear response. An overview of these differences is provided in Table 2.

When polymer composites are exposed to elevated temperature regimes, particularly regions approaching or exceeding T_g_, the matrix transitions from a glassy state to a viscoelastic state. While standardized shear test methods and flexure-based approaches provide well-established baselines under ambient conditions, their validity can be compromised under conditions of elevated temperature exposure. These methods rely on fundamental assumptions of uniform stress distribution, elastic material response, and sustained composite action, which may no longer hold as the matrix softens, and the fiber–matrix interface degrades as a result of elevated temperature exposure. Since standardized test methods are derived from linear elastic beam theory, assuming small deformations, constant geometry, and uniform material properties, once these assumptions are violated, the stress and strength calculations prescribed by the standards may no longer be valid. Under elevated temperature exposure, degradation of the polymer matrix and fiber–matrix interface occurs concurrently with matrix softening, leading to a pronounced reduction in shear stiffness (e.g., G_12_) and potential dimensional instability/change. Because current test methods assume uniform specimen properties, these changes can invalidate the different stress state assumption in the standards [86]. Additionally, mismatches in the coefficients of thermal expansion (CTEs) between the specimen and the loading fixtures or grips can introduce unintended mechanical stresses, misalignment, or grip loosening [87]. These effects lead to non-uniform load introduction and further violate the assumption of uniform stress and strain fields, thereby compromising both the accuracy and repeatability of the measured properties. Thermal gradients developed across the specimen thickness [88] exacerbate these issues.

As temperatures approach the glass transition temperature (T_g_), localized matrix yielding, interfacial slip, and resin flow can also lead to redistribution of stresses away from the intended shear-dominated region, potentially invalidating nominal strength calculations. At higher temperatures, additional experimental challenges can arise, including notch blunting in Iospescu specimens, grip slippage in in-plane shear tests, and loss of arch action in short-beam shear configurations. Thermal gradients through the specimen thickness, particularly under rapid heating or thermal cycling, can further violate assumptions of homogeneous temperature fields. Consequently, measured reductions in shear strength may reflect a combination of true material degradation and test-induced artifacts. These limitations, as summarized in Table 3, underscore the need for careful interpretation of shear data collected under elevated temperature conditions, and thus, there is a need to be cautious about the direct comparison of results obtained from different test configurations or exposure protocols without assessing effects on the viability of the test itself.

From a mechanics perspective relevant to shear response, thermal exposure of FRP composites may be broadly characterized into 3 regimes: (i) temperatures well below T_g_, where elastic response-dominated shear transfer prevails; (ii) temperatures in the vicinity of T_g_ characterized by stiffness degradation, matrix softening, and progressive interfacial slip; and (iii) temperatures above T_g_ but below T_d_, where loss of load carrying capability occurs primarily through matrix flow and stress redistribution. This then provides the operational subset of the regimes listed in Figure 3, and it should be emphasized that scenarios involving matrix decomposition, severe charring, or combustion fall outside these regimes and are not considered herein.

## 3. Effects of Uniform Thermal Loading on CFRP and GFRP Composites

The inherent differences in the material properties between the two main categories, such as the comparatively superior stiffness and durability of CFRP [89] and good specific resistance and low thermal conductivity [90] of GFRP, influence their performance during thermal exposure. A critical distinction also arises from their different CTEs, which result in varying thermal strains between the fibers, matrix, and the fiber–matrix interphase during exposure to elevated temperatures [33,34]. Since the shear response of composite materials is primarily governed by the integrity of the matrix and the fiber–matrix interface a comparison of the shear behavior provides the most direct means of understanding the differences between these materials in terms of thermal aging.

Although temperatures approaching T_g_ are examined in this review to elucidate degradation mechanisms and transitions in failure modes, such conditions do not represent typical design targets for structural FRP systems. In practice, service temperatures are generally maintained well below T_g_ to preserve stiffness, strength, and interfacial integrity with near-T_g_ exposure primarily relevant to abnormal or transient thermal events.

### 3.1. Interlaminar Shear Response

**Figure 10:**
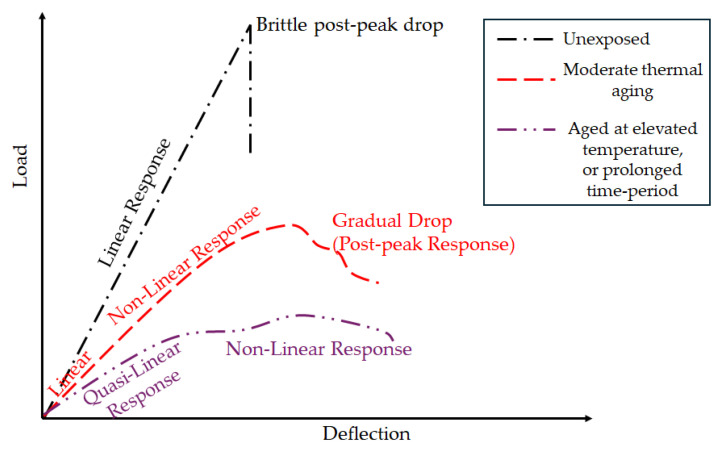
General response of load displacement curve under thermal load.

The load–displacement (L-D) curve serves as a crucial tool for evaluating the mechanical response of composite materials under different loading conditions [91], providing critical insights into the composite’s behavior under thermal load. The L-D response of an ILSS specimen can be described in three phases—linear, nonlinear, and post-peak behavior [92]—as shown schematically in Figure 10. For a thermally unaged specimen, the L-D curve increases linearly up to the peak load [93,94] and then drops suddenly [24,93]. In contrast, thermally aged specimens exhibit an initial linear region [43,92,93,95], with deviations from linearity with increasing load [94] and temperature [92]. With an increase in temperature, post-peak behavior gradually shows a plastic response due to the transition of the resin response from a glass to leathery state [92]. Thus, at lower temperatures of thermal aging, post-peak drop is reported to be brittle [95], while with an increase in temperature, the drop is gradual [24,93]. However, Tan et al. [96] observed that both thermally unaged and aged specimens (80 °C for 28 days) displayed a linear increase up to a peak, followed by a sharp drop, irrespective of the level and extent of aging. The discrepancy in the test results compared to the behavior reported by other researchers can be attributed to an increase in level and extent of post-curing in specimens, suggesting that higher degrees of cure contributed to increased brittleness [97]. For both fully and partially cured systems, the peak load decreases progressively with rising temperature [43,92,96]. The transition from linearity (governed by the elastic response of the matrix and interphase [94]) to nonlinearity, driven by viscoelastic behavior [43,92,94], is influenced by thermal exposure [43] or thermal aging time [93] and is associated with shifts in the failure mode [93] with mechanisms of matrix softening, crushing, and delamination [92]. For unaged specimens, the stress–strain curve also shows a linear increase up to the peak stress, followed by a sudden drop [24]. In contrast, specimens aged at elevated temperatures display either initial linearity [24] or quasi-linearity [98], but with a progressively decreasing slope during the early stage, with an increase in temperature [24,98]. Beyond a critical (peak) stress level, all curves transition into a nonlinear regime due to damage mechanisms such as delamination [24,98] and micro-buckling, which intensify with increasing temperature [24].

The nature of the stress–strain behavior has also been correlated with the T_g_, with specimens aged below T_g_ typically exhibiting a sudden post-peak drop indicative of brittle failure, whereas specimens aged above T_g_ display a more gradual decline reflecting ductile or plastic-like deformation [24]. This shift in material response near T_g_ is also shown by a progression in physical failure modes [3,94,99], which can be categorized into three main types: brittle failure, progressive failure, and multiple shear or extensive delamination [93]. This progression can be linked to three distinct temperature regions as proposed by Bazli et al. [25] and encompassed in the general structure of Figure 2.

1. Region 1 (T < T_g_): In CFRP, mechanisms include interlaminar delamination [24], kinking in the loading direction [43] and crenulations and radial patterns at the fiber ends [100]. Similarly, for GFRP, the mechanisms of delamination [92,101], compression failure [92] and horizontal shear failure at the midspan along the neutral axis [102] are noted. In transversely loaded GFRP specimens, the onset of localized flexural cracks with crack-induced delamination is observed, with the failure mode becoming more intense with an increase in temperature but essentially remaining unchanged [92].
2. Region 2 (T ≈ T_g_): This stage serves as the onset of progressive failure modes. As the temperature approaches the glass transition, matrix softening allows for more ductile failure modes along with yielding and extensive loss of the matrix [100]. For CFRP specimens, fracture surfaces become rougher, with evidence of plastic deformation (e.g., wider bulges), stepwise bending [43], interlaminar delamination [98], and fiber bridging [43]. Similarly, in GFRP specimens, the failure in Region 1 is highlighted with an increase in microcracks, with these inducing delamination with increasing temperature [92].
3. Region 3 (T_g_ < T < T_d_): This is the third region, in the rubbery state above T_g_. For CFRP, the matrix offers little support, leading to extensive surface degradation with a number of loosely attached fibers [103] and interlaminar delamination accompanied by apparent fiber bridging and buckling failure [24]. Similarly, for GFRP, higher indentation and a lower number of ply delaminations due to increased viscoelasticity [92] are reported, and in the transversely cut specimen, more severely distributed flexural cracks with crack-induced delamination than at lower temperatures [92] are observed.

Aging duration and temperature act synergistically, meaning prolonged thermal exposure, even at sub-T_g_ temperatures, can induce failure modes typically associated with higher temperature regimes [104,105]. Liu et al. [106] showed that interfacial debonding, which was evident on the surface of the specimen after aging for 1000 h at 200 °C, was seen substantially earlier at 600 h at 250 °C of thermal aging. Similarly, Li et al. [107] reported that the thermal aging for 2000 h at approximately 0.66 T_g_−0.82 T_g_ produced microcracks and microvoids, while exposures beyond 3000 h led to fiber pull-out, transverse tensile failure, fiber fracture by buckling, and inter-ply delamination. Similarly, Akay et al. [93] showed that unaged specimens fail by brittle transverse tensile fracture, but long-term thermal exposure shifts the failure mode towards fiber buckling accompanied by delamination. These observations highlight that the time and temperature control not only the rate of degradation but also the progression and nature of failure. However, extending the timescale over a very short range may have a negligible impact on FRP behavior due to the dominance of temperature over duration. For instance, increasing the time of thermal aging from 10 to 60 min at any given temperature up to 0.4 T_g_ was shown to have a negligible impact, whereas increasing the temperature toward T_g_ resulted in a substantial ILSS reduction of 25–30% [108]. A similar result was shown by Barile et al. [105]. It can be seen that at sub-T_g_ levels, short-term exposures mainly capture the immediate, reversible effects of physical aging, while the irreversible effect of chemical aging is more significant over prolonged durations [109].

When subjected to increased time and temperature of thermal aging, the specific response is shaped by the competing effects of post-curing and degradation and is dependent on the complex interplay of experimental conditions, material characteristics, and underlying physical and chemical properties [24,43,98,107,110,111,112]. For fully cured composites, strength starts to decrease gradually due to deterioration of the matrix and fiber–matrix interface starting from the initial exposure condition in both CFRP [13,98] and GFRP [95]. In contrast, for composites that are not fully cured, initial thermal exposure can temporarily increase strength due to additional crosslinking, the release of residual stresses, and the closure of microcracks, leading to post-curing [3,13,29,104,106,113,114] as well as secondary curing and densification of the polymer chains [96,114,115,116]. A similar response in terms of an increase in strength has also been reported for GFRP [95,102,117], with researchers having reported a maximum increase of ILSS of 27% at temperatures up to 232 °C even after 72 h [3] and an increase of ILSS of up to 89% for specimens aged for 400 h at 200 °C [106].

While the conventional model of post-curing followed by degradation offers a useful framework, certain GFRP systems display more complex, non-linear thermal responses. In vinyl ester and unsaturated polyester-based GFRP, for example, an initial increase in ILSS strength at low temperatures was absent; instead, strength declined linearly up to 40 °C. Between 40 °C and 60 °C, however, ILSS rose by approximately 10% in the polyester and 6% in the vinyl ester before entering a final decline with further heating. Notably, at 200 °C, both the polyester and vinyl ester composite retained around 90% of the initial baseline strength, attributed to strong fiber–matrix adhesion and fiber bridging [118].

However, such strength gains due to post-cure are transient. As time or temperature increases degradation phenomena such as chain scission, thermal oxidation, microcracking, and the build-up of high internal stresses, the generation of radial tensile and axial compressive stresses [103] also begin to dominate, leading to a decrease in strength. As the temperature rises, the time required to reach the peak ILSS shortens, and post-peak deterioration becomes more severe, likely due to the loss of cohesive strength at the fiber–matrix interface, as higher temperatures accelerate damage mechanisms at this level [3,106]. For instance, a GFRP specimen aged at 60 °C reached its peak ILSS at 56 days, while the same specimen aged at 80 °C peaked earlier at 28 days. After 224 days, deterioration was higher at 80 °C (13% loss), whereas the 60 °C specimen still retained 5% strength [117]. This illustrates the synergistic effect of time and temperature, where higher temperature leads to deterioration in a shorter period and vice versa. A similar trend was reported for CFRP, where the deterioration of the CFRP specimen aged for 11,000 h at 232 °C was reported to be 8.1 times slower than the specimen aged for 2500 h at 288 °C [107]. This time–temperature synergy was evident not only under conditions of elevated temperature but also under radiant heat flux, as shown by Mouritz and Mathys [119], who reported that at a lower heat flux (50 kW/m^2^), it took 500 s for a complete deterioration, while at 325 s, the retention of the specimen was 25%. This indicates that, although post-curing increases strength, deterioration due to the synergistic effect of time and temperature will ultimately dominate post-curing effects, causing strength to decrease.

The glass transition temperature (T_g_) is arguably the most critical parameter governing the competition between post-cure and deterioration. It serves as a distinct threshold, below which degradation kinetics are relatively slow and post-curing effects can be prominent. For example, in a study of CFRP, extended thermal exposure in the range of 50–100% of T_g_ results in minimal deterioration (~17%) even after exposure for different time periods up to 2160 h; however, strength loss accelerates sharply beyond T_g_, reaching up to 90% within 72 h at approximately 145% of T_g_ [13]. A similar result was also reported by Rathore et al. [120] for GFRP, where reductions of 10%, 15%, and 46%, were observed for specimens exposed to temperature levels of 0.62 T_g_, 0.8 T_g_, and 0.98 T_g_, respectively.

The reason for the deterioration of the specimen’s strength at temperatures near or above T_g_ is that the polymer matrix softens to a rubbery state, allowing polymer chain relaxation and causing an exponential increase in chemical degradation rates, which leads to immediate and severe strength loss. Thus, composites with a lower T_g_ demonstrate inferior thermal stability, showing rapid deterioration at lower temperature thresholds due to higher internal stresses and increased thermal expansion mismatch [121]. For example, composites with T_g_ values of 177 °C and 192 °C exhibit significantly different strength reductions (67% vs. 53%) when tested at 200 °C [43]. Similar results were shown by Li and Xian [111] as well. These findings highlight that since T_g_ is an important property of the composite matrix and also a critical threshold, the composite matrix should be selected based on a T_g_ level appropriate to its service conditions to ensure long-term thermal stability. To mitigate early degradation arising due to lower T_g_, post-curing can be employed, thereby enhancing thermal stability and delaying the onset of temperature-induced deterioration. However, the benefits of post-curing are highly dependent on processing conditions, and improper application of temperature or duration of thermal load may lead to residual stress or compromise long-term mechanical performance.

Although exposure to elevated temperature levels can raise the T_g_ [122,123,124], these higher temperatures can also induce residual stresses due to thermal expansion mismatch between fibers and the matrix, as well as matrix shrinkage during cooling [124], both of which can result in a reduction in mechanical properties. Importantly, this degradation from microcracks occurs only after post-curing and is not related to pre-cure exposure [125]. Studies have shown a non-linear relationship between post-cure temperature and ILSS, where strength decreases at lower post-curing temperature, likely due to residual stress-induced microcracking [124,126]. It is noted that after reaching a specific optimum post-cure temperature, strength continues to increase with further increase in post-cure temperature. For instance, the ILSS of CFRP decreased by 27% up to a post-cure temperature of 350 °C then rose by 18% at a post-cure temperature of 375 °C [124]. Similarly, Kumar et al. [126] reported on the effect of the post-curing temperature of a GFRP composite over 12 h, showing that at the highest temperature (140 °C), ILSS increased sharply, by about 25%, at a rate of 1.24 MPa per hour up to 6 h, after which it reached a plateau due to the rapid development of interfacial crosslink density, which eventually saturated with time. In contrast, post-curing at 110 °C resulted in a significantly slower strength gain, while specimens cured at 80 °C exhibited an immediate plateau, indicating that these lower temperatures were insufficient to advance the cure state substantially [126]. These findings underscore the importance of carefully selecting post-cure temperatures to exceed a critical threshold, thereby ensuring that the benefits of post-curing outweigh the detrimental effects of residual stress and microcracking, ultimately achieving a net gain in mechanical performance.

However, even after selecting the optimum post-curing temperature or sticking to the manufacturer-recommended cure (MRC) cycle, deviations in temperature may be unavoidable, which can significantly impact the mechanical strength of composite materials. Alavi-Soltani et al. [123] reported that specimens cured at a temperature of 149 °C, which was lower than the optimal temperature of 177 °C for post-cure, led to a 10% lower ILSS due to decreased crosslinking and weaker bonding between plies.

As the specimen approaches its T_g_, matrix softening reduces load transfer across the fiber–matrix interface, making the selection of an appropriate matrix critical for maintaining a strong interface. A stronger interface not only preserves structural integrity but also resists delamination and delays catastrophic failure beyond the contribution of the fiber alone. Studies comparing specimens with the same carbon fibers but different matrices [43] and vice versa [94] demonstrated that variations in interface strength, largely controlled by the matrix, resulted in noticeably different peak loads, with stronger interfaces producing a higher ILSS. Vieira et al. [95] showed that under identical thermal conditions, for glass fiber-reinforced materials, a vinyl ester matrix led to a gradual decline in ILSS, whereas a phenolic matrix provided an initial increase before degradation.

While the ILSS is predominantly a matrix-dependent property, the intrinsic thermal stability of the reinforcing fibers also plays a crucial role in its degradation at elevated temperatures [111]. Carbon fibers have a very high modulus and low breaking strain, while glass fibers possess a lower modulus but a greater extension to failure [110]. These fundamental differences in mechanical and thermal properties lead to different degradation responses. A comparative study by Aklilu et al. [110] investigated the ILSS of CFRP and GFRP specimens with equal fiber volume fractions after exposure to 55 °C. At ambient conditions, the CFRP and GFRP specimens exhibited an ILSS of 50.77 MPa and 43.36 MPa, respectively. After thermal exposure, the CFRP strength decreased to 32.34 MPa (64% retention), whereas the GFRP strength dropped to 26.58 MPa (61% retention). These results highlight that CFRP exhibited a higher ILSS at room temperature due to the presence of carbon fibers; however, the retention at elevated temperatures showed only marginal differences between the two systems, as it is mainly governed by matrix plasticization or degradation at the fiber–matrix interface. Akay and Spratt reported that composites with a higher fiber volume fraction initially show higher levels of ILSS, but prolonged thermal aging induces greater interfacial stresses, accelerating microcracking and reducing ILSS [112].

Shaoquan et al. reported that unidirectional (UD) laminates demonstrate substantially better ILSS retention compared to multidirectional (MD) layups after thermal exposure [114] because when the shear load aligns with the fiber direction, the continuous, aligned fibers efficiently carry the load, making the laminate less susceptible to degradation. In MD laminates, the presence of off-axis plies serves as an initiation site for matrix-dominated damage, such as off-axis cracking and fiber–matrix debonding, even under low stress levels. For example, a study comparing both UD [0_8_]_S_ and MD [45/0/–45/90]_2S_ specimens showed that the UD specimen consistently maintained 35–50% higher strength after 1000 h of aging at 200 °C, confirming the superior stability of fiber-dominated layups [114]. This damage can propagate rapidly between differently oriented layers, causing premature structural failure [114] by creating low-energy paths for crack propagation that are exacerbated by thermal degradation [113]. The critical influence of fiber orientation is further demonstrated by the role of loading direction. Studies on GFRP composites show that specimens tested longitudinally (along the fiber direction) exhibit a substantially higher initial strength and retain greater load-bearing capacity at elevated temperatures compared to transverse specimens [92]. Manalo et al. [92] reported that a longitudinally loaded specimen exhibited a strength decrease from approximately 55 MPa at room temperature to 8 MPa at 100 °C, whereas transverse specimens dropped from 21 MPa to nearly zero over the same temperature range. This difference arises because the longitudinal properties rely on the fibers, which are thermally stable, whereas the transverse properties rely heavily on the matrix, which softens near its T_g_ and thus exhibits weak performance with an increase in the level of thermal aging [96].

ILSS is highly dependent on the experimental conditions, and hence particular focus must be paid to the distinction between testing specimens after cooling (residual strength) versus testing in situ at elevated service temperatures. Tan et al. [96] and Lv et al. [127] reported that room temperature testing can be non-conservative, as it fails to capture the immediate and severe effects of matrix softening and accelerated strength degradation at high temperatures. In fact, specimens may even exhibit increased strength when tested at room temperature due to post-curing effects, while the same materials suffer significant strength loss when tested at elevated temperatures [127], with the difference being more pronounced with longer durations of thermal exposure. Sun et al. [124] investigated the effects of composite processing history and subsequent mechanical performance by first exposing a CFRP specimen to different temperatures for post-curing (315 °C to 375 °C) and noted the difference in strength between tests conducted at room temperature after cooling and under in situ (400 °C) conditions. The strength of the specimen when tested at room temperature was found to be higher by a minimum of approximately 55% than when tested at in situ (400 °C) conditions. ILSS initially decreased with increasing post-cure temperature from 315 °C to 350 °C and then increased moderately with further increases in the post-cure temperature. This contrast arises because the matrix is rigid and brittle at low temperatures but becomes viscoelastic at elevated temperatures due to polymer chain relaxation, leading to reduced strength, more ductile fracture behavior [127], and pronounced tearing and rougher fracture surfaces compared to the room temperature test [96].

Beyond the details of environmental exposure, the measured ILSS of a composite is highly sensitive to loading rate or crosshead speed [100]. This behavior is a direct consequence of the polymer matrix’s viscoelastic nature. At a constant temperature, a higher crosshead speed results in a greater apparent ILSS because the reduced testing time limits damage mechanisms such as sub-critical crack growth and restricts the time available for molecular-level viscoelastic relaxation within the polymer chains. For example, at a loading speed of 1 mm/min, strength reductions relative to ambient conditions can exceed 80.57% at 50 °C and 93.25% at 100 °C, whereas at a speed of 1000 mm/min, the corresponding reductions are approximately 30% and 80%, respectively [100].

### 3.2. In-Plane Shear Response

In-Plane Shear (IPS) tests characterize the shear response within the plane of a lamina. While it is also a matrix-dominated property like the ILSS, the mechanical response and failure mechanisms under thermal loading are fundamentally different and, in many ways, more complex, with complexity amplified at elevated temperatures through a shift in the position of the neutral axis and initiation of shear-induced delamination, resulting in progressive degradation of the material’s load-bearing capacity as temperature increases [128]. A review of the literature shows that the temperature-dependent behavior of load-displacement and stress–strain curves for in-plane shear response is not consistent across the literature [98,128,129,130,131,132,133,134]. Bhargava and Zehnder [129] reported an initial linear region followed by a nonlinear transition with increasing load and/or temperature, regardless of the level of thermal aging. Similar responses were also reported for L–D curves obtained from 10° off-axis tensile tests [130], and Iosipescu tests [131], as well as from stress–strain curves derived from Iosipescu testing [132]. In contrast, Correia et al. [133] reported that 10° off-axis GFRP specimens exhibited a linear L-D response up to failure, even when aged at temperatures as high as 250 °C, with only slight reductions in stiffness prior to failure. In contrast to both the previous sets of observations, other researchers reported non-linear stress–strain responses for both CFRP and GFRP at room and elevated temperature levels through test methods such as short-beam shear [128], ±45° tension [98] for CFRP, and ±45° tension for GFRP [134]. This observation suggests that the onset of linear and non-linear behavior in both CFRP and GFRP may be influenced by the specific testing methodology employed. Although the particular reason for the observed non-linear effect is not completely known [135], it is assumed that the difference in Poisson’s ratios of the fiber and matrix, the lateral deflection of the imperfect or misaligned fiber [136], and the effects of microcracks [135] could be the potential reasons.

**Figure 11:**
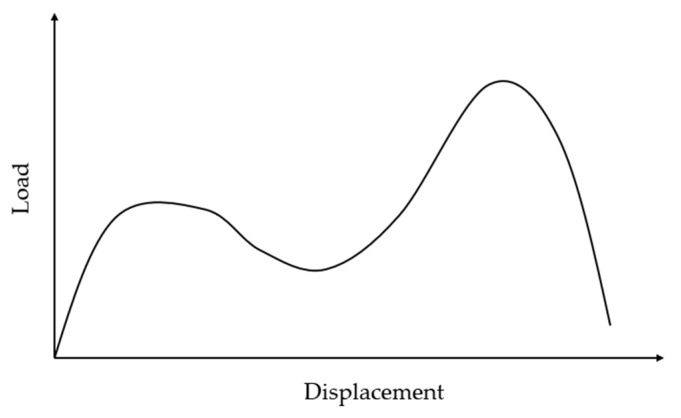
Dual-peak response of load–displacement curve due to reorientation of fibers.

While the reviewed literature consistently shows that unaged CFRP [98,128,129,137] and GFRP [130,131,132,133] composites exhibit higher peak loads than thermally aged counterparts, the post-peak failure behavior is controlled by softening of the matrix caused by an increase in temperature. At lower temperatures, failure is characteristically brittle, whereas at elevated temperatures, the failure mode transitions to a more ductile response [130,131], as also seen in the behavior during the ILSS test. This trend is particularly pronounced at temperatures exceeding the matrix’s T_g_, where the load–displacement curve may exhibit a distinct plateau indicative of significant plastic deformation [131]. An interesting exception to the commonly noted response was reported by Bhargava and Zehnder et al. [129] at a temperature of 315 °C (for CFRP with a T_g_ of 358 °C) where the L-D curve initially increases to a peak, then decreases, and subsequently rises sharply again, as shown schematically in Figure 11. This dual-peak response is due to reorientation of fibers within the sheared gauge section after the initial failure of the severely weakened matrix at high temperature. Once reoriented, the fibers begin to carry the load predominantly in pure tension, producing a second, often higher, peak load before ultimately failing in rupture [129]. It is emphasized that this failure mode is different from that observed in ILSS or flexural tests under similar thermal conditions.

The failure mechanisms for in-plane shear loading evolve systematically with temperature and can be categorized into three distinct regions relative to the material’s T_g_ using the framework proposed by Bazli et al. [25].

- Region 1 (T < T_g_): In this phase, the matrix is in its rigid, glassy state. For CFRP, Iosipescu test specimens show the formation of shear cracks that propagate along the direction of the fiber [129], while ±45° off-axis test specimens show the fracture line propagating along the fiber direction [3,98,138], resulting in delamination [3,44,98,137,138,139], fiber bundle pull out [98,137,138], and release of tensile stress with resin wrinkles [98]. For GFRP, specimens tested using a 10° off-axis test show the fracture parallel to the roving’s direction, without any breakage of fibers except within surface mats [130,133], with mechanisms being independent of temperature [130], while Iosipescu test specimens show shear failure in the central section of the specimen with vertical fracture surfaces [131,132], along with rupture of the matrix and of surface mats [131].
- Region 2 (T ≈ T_g_): In this temperature range the matrix begins to soften, allowing for greater plastic deformation and more complex failure interactions. For CFRP, the specimen tested using the 45° off-axis test shows damage propagating along the 45° axial direction, leading to more pronounced interlaminar cracks [138], delamination [98,138,139], and fiber pull-out [137], and in some cases, reorientation of fibers further contributes to loss of shear strength [44]. In composites with woven architectures, the transverse fiber region plays a crucial role in bridging cracks and holding the specimen together even after significant matrix damage, preventing catastrophic failure [139]. For GFRP Iosipescu test specimens, geometric stress concentrators such as the V-notches become focal points for damage initiation [131]. In this thermal regime, the softened matrix is unable to sustain the localized compressive stresses, leading to a premature failure mode, with local crushing at the edge of the notched area [131,132] and ply delamination [131]. For the specimen tested using a 10° off-axis specimen, failure is noted to be similar to that seen in Region 1, since the failure mode is independent of temperature [130].
- Region 3 (T > T_g_): The matrix is in a rubbery, viscoelastic state in this thermal regime. For CFRP, in comparison to the previous regions, matrix adhesion to fibers was reported to be higher, leading to increased interfacial bonding at elevated temperature [39]. Debonding and interlaminar cracks are expected to be more critical in this region [138]. For GFRP, the prevailing failure mode is similar to that seen in Region 2, with localized crushing at the edges of the notched region, initiated by the high stress concentrations imposed by the loading blocks [131,132], along with delamination and premature failure [131].

The competing effects of temperature and time of thermal aging noted in the previous discussion on ILSS specimens is also observed in IPSS specimens. A slight initial increase in IPSS can be noted at moderate temperature levels attributed to improved interfacial bonding [140] and the relaxation of residual stresses at the fiber–matrix interface caused by differences in their CTE, thereby promoting better fiber–matrix adhesion, before the onset of degradation in both CFRP [39,98] and GFRP [118]. Additionally, degassing of the water potentially present in the specimen and formation of graphite crystals during stretching, graphitization, and increment of fracture strain of the fiber at elevated temperatures are also found to increase the strength between the fiber and matrix in CFRP [141]. However, this beneficial effect is not universal, with decreases in IPSS of both CFRP [111] and GFRP [22,132,133,134] being reported because of the rapid softening of the resin at elevated temperatures, which leads to the early onset of failure modes such as the separation of layers and the sliding of the fibers [3]. During this process, thermal residual stress causes the initiation of small cracks in the matrix and in the interphasial regions. These microcracks also change the location of initial damage, path, and ultimate strength of the composite [142].

Glass transition temperature plays a crucial role in governing the deterioration of IPSS. Quantitative assessments indicate that significant strength losses of CFRP specimens accrue when the thermal aging temperature approaches T_g_, with reductions in the range of 19–44% observed between 0.6 T_g_ and 0.85 T_g_ [139]. Similarly, in the case of GFRP, Mazzuca et al. [132] reported modulus reductions exceeding 61% at temperatures close to T_g_, compared with less than 7% losses at intermediate temperatures. At even higher temperatures, because of the rubbery state of the matrix, reductions approaching 90% were reported in GFRP specimens, highlighting the matrix-dominated nature of the property [130,133,143] and confirming that IPSS, like ILSS, serves as an early indicator of thermal damage. A comparative analysis across multiple datasets reveals a critical threshold of temperature of approximately 70 °C for CFRP, below which the IPSS remains largely stable, and degradation rates are minimal [98]. Above this threshold, accelerated softening of the matrix and weakening of the fiber–matrix interface drive rapid declines in shear performance [98].

The duration of thermal aging is a principal determinant of both the rate of property degradation and the governing failure mechanisms. The transition from one failure mode to another over time is a clear indicator of the material’s evolving structural response under thermal stress. A study by Madhukar et al. [140] showed that CFRP specimens aged at 316 °C for up to 500 h experienced minimal changes in fracture surface morphology and negligible strength loss, indicating that the composite’s primary load-bearing capacity remained intact. However, when the time of thermal exposure was extended to 1000 h, a fundamental shift occurred with the fracture path transitioning entirely from cohesive failure within the matrix to interfacial debonding, resulting in a loss of strength of approximately 13%.

Specimen geometry, such as width, is noted to have a significant effect on the strength determined at both room and elevated temperature levels, with wider specimens often yielding higher values due to a more uniform stress distribution for the 45° test, with reduced influence of edge effects and hence a more uniform stress field [144]. While the preparation of a test specimen is always important, aspects such as edge effects and damage are of special impact in IPS-type specimens, where even differences in edges accruing from waterjet cutting versus mechanical machining have been shown to significantly alter test results by introducing different levels of edge damage [145]. Additionally, different testing methods also lead to different reported in-plane shear strengths. Gentz et al. [144], for example, reported that at 315 °C, the ±45° test failed at a lower shear stress (37.3 MPa) due to the onset of intralaminar damage than the Iosipescu test, which showed a failure strength of 59.9 MPa because of the creation of a biaxial stress field in the case of the ±45°. A comparative study by Rosa et al. [131] on GFRP specimen revealed that while the degradation pattern with increase in temperature was similar, the rate of strength loss measured by the Iosipescu test was significantly faster (88% at 180 °C) than that measured by the 10° off-axis test because of the presence of tensile stresses in the 10° off-axis test, which are less susceptible to elevated temperature degradation. This highlights a critical challenge in that the lack of a single, universally accepted test standard makes direct comparison of absolute IPSS values between studies using different methods extremely difficult if not invalid in some cases.

In-plane shear strength has been reported to be more sensitive to elevated temperature exposure than the corresponding shear modulus [3,131,137,139] and stiffness [131,133] in both CFRP [3,133,137,139] and GFRP [131]. This difference in thermal response is rooted in the distinct roles of the matrix and fibers in defining each property. The shear behavior of a composite is primarily dominated by the polymer matrix, which is highly susceptible to thermal degradation, particularly as it approaches its T_g_ [128]. As a result, the IPSS, which is a matrix-dominated property, experiences a rapid decline past a critical threshold level with increasing temperature. In contrast, while the shear modulus is also influenced by the matrix [128], it is also dependent on the contribution of the reinforcing fibers [146], which are significantly less susceptible to temperature increases, resulting in the modulus being less sensitive to the thermal aging of the matrix [128]. However, a few researchers have reported a greater deterioration in shear modulus than in shear strength. Gentz et al. [144] observed a 34% reduction in shear strength for CFRP aged from room temperature to 315 °C, while the shear modulus decreased by 88% over the same range. Similarly, Chow et al. [134] reported for GFRP that shear strength decreased by 93.11% at 110 °C, whereas the modulus degraded by 97.49%. However, sometimes these apparent contradictions can be the products of experimental artifacts or specific methodological designs, as Gentz et al. [144], for example, reported that in their experiment, the trapped heat from strain gauges insulated with silicone layers caused the matrix to approach its glass transition, thereby artificially exaggerating the reduction in the modulus. Furthermore, measured properties can also be highly dependent on the spatial distribution of thermo-oxidative damage. In a study on unidirectional graphite/PMR-15 composites aged at 316 °C, Madhukar et al. [140] found that the in-plane shear modulus was unaffected by aging for up to 1000 h because the test specimens were machined from the interior of larger composite panels, which were exposed to the temperature rise before the specimens were cut from areas where the material was minimally degraded. Optical analysis confirmed that the thermo-oxidative damage, such as matrix microcracking, was confined to a thin surface layer near the exposed edges of the larger plates. These discrepancies underscore the need for careful interpretation of experimental data and for standardized testing protocols that can more reliably capture the true thermo-mechanical response of FRP composites.

### 3.3. Shear Response from Flexural Loading

Flexural strength is defined as a material’s ability to resist bending until failure occurs. This failure is a direct result of the complex stress state experienced during flexural loading, where a specimen is subjected to compressive, tensile, and shear stresses [147]. Flexural failure often initiates in the matrix and interface regions where shear stresses are concentrated [3]. Since FRP systems rely heavily on the resin and interface to preserve structural cohesion, and because flexural loading places significant demand on these areas (resin and interface), the assessment of flexural strength serves as a key measure of composite performance and durability under thermal stress [3].

At a macroscopic level, for both CFRP and GFRP, the load–displacement (L-D) and stress–strain profiles in flexure for FRP composites mirror the behavior observed through ILSS tests. The response seen in the L-D curve can be divided into three regions—linear, non-linear, and post-peak stage [92]—as shown schematically in Figure 10. At room temperature, the behavior is linear, reaching the peak load followed by a sudden drop, indicating brittle failure [92,148,149]. As the temperature increases, the response enhances nonlinearity [25,92]. With an increase in temperature, post-peak behavior gradually shows a plastic response due to the transition of resin from a glassy to a leathery state [92], leading to softening [149]. Thus, at lower temperatures of thermal aging, post-peak drop is reported to be brittle, while with an increase in temperature, the drop transitions to be more gradual [92,150]. Manalo et al. [92] reported that the L-D behavior of transversely loaded specimens is similar to that of the longitudinally loaded specimens. This degradation is not solely a function of temperature but is also affected by the period of prolonged thermal aging in both CFRP [151] and GFRP [25] composites. As both the duration and temperature of thermal aging increase, so does the level of deterioration. During this process, thickness becomes a key factor in determining the extent of material deterioration and how far heat-related damage penetrates the laminate [25]. Despite the similar nature of L-D profiles, thicker specimens consistently exhibit a higher peak load than thinner specimens at both room temperature and elevated temperatures [8,25] because thick specimens show more resistance to bending moments and carry higher loads [8]. Bazli et al. [25] reported that for GFRP specimens, an increase in thickness resulted in a significant increase in peak load. Unidirectional specimens at 2 mm and 5 mm thickness with fiber weight fractions of 70.5% and 71.2%, respectively, showed peak loads of 870 N and 5500 N, respectively, indicating a relationship, as expected, of peak load being dependent on the square of specimen thickness. It is of interest to note that the peak load measured after 120 min of exposure at 300 °C was 69 N for the 2 mm thick specimens and 440 N for the 5 mm thick specimens, with both resulting in a 92% decrease.

Failure mechanisms are noted to evolve with changes in thermal loading levels in both CFRP [3,43,112,148,152,153] and GFRP [25,95,149,153]. In CFRP, the failure mechanism is governed by the stress level that the specimen can withstand before delamination when subjected to elevated temperature, with the specimen able to withstand higher stress levels failing in tension, whereas those at lower levels failed in shear with more complex and interacting modes being seen between these extremes, with failure mode dependent upon the temperature of exposure [152]. The evolution of failure mechanisms based on level of thermal exposure can be categorized into three distinct regions relative to the material’s T_g_ following the same framework used for ILSS and IPSS.

- Region 1 (T < T_g_): In the rigid, glassy state, failure is typically brittle and fiber-dominated in both CFRP and GFRP [147]. CFRP typically fails due to compressive fiber kinking [43,147,148] or micro-buckling [147,148] on the upper surface, often accompanied by fiber rupture on the lower tensile surface [43,148,153] and localized microcracking [3]. GFRP composites primarily experience fiber tensile failure at midspan at the lower surface [25,149,153], followed by interlaminar delamination [95,149]; fiber kinking and micro-buckling [147]; and other brittle phenomena, such as river line cracking, splitting, fiber pull-out [154], and fiber fracture occurring in a piecewise fashion [149].
- Region 2 (T ≈ T_g_): As the matrix softens, failure modes in both composites shift to resin-influenced modes. For CFRP, the failure shifts toward shear-mode micro-buckling with significant plastic deformation [148] and tensile failure at the bottom surface [153]. GFRP also shows local tension failure at the bottom surface [153] and shear failure [25] along with compressive buckling [8,25,149].
- Region 3 (T > T_g_): In the soft, rubbery state, the matrix becomes the definitive weak link for both materials, with delamination being the most prevalent failure mode as the matrix can no longer provide adequate ply cohesion or support against fiber buckling for both CFRP [3,153] and GFRP [25,95,155]. For CFRP, micro-buckling on the top surface at the loading point and multiple inter-fiber cracks are observed due to matrix failure but without plastic deformation [43], and for the GFRP, multimode failure is observed under the loading nose [153].

When a composite material is subjected to elevated temperatures, its mechanical strength exhibits one of two general trends, governed by the competition between post-curing and degradation. This duality is described in two stages: the first is the consolidation phase, and the second is the degradation phase [26]. The consolidation phase is characterized by enhanced molecular mobility and crosslink density that boosts T_g_ and mechanical strength. During this stage, incompletely cured composites can experience additional crosslinking due to thermal exposure, leading to an increase in flexural strength in both CFRP [3,13,26,127,152,156,157,158] and GFRP [118]. As thermal exposure continues, the dominant mechanism shifts from consolidation to degradation. This phase is marked by a progressive loss of mechanical integrity. A primary cause is the mismatch in the CTE between the reinforcing fibers and the polymer matrix [159]. This disparity generates significant residual stresses, which can lead to the formation of microcracks at the fiber–matrix interface [159] and degradation of the fiber–matrix bond [3]. These microcracks act as pathways for atmospheric oxygen [159], which accelerates thermo-oxidative degradation and plasticization of the resin [26,27]. Simultaneously, prolonged heat exposure can cause scission of polymer chains, a process known as chain degradation [27], which further contributes to the loss of mechanical properties in both CFRP [3,13,29,156] and GFRP [15,22,118,149,150,154,155]. While the level of temperature controls the potential severity of degradation, the rate and extent of property loss and failure initiation are governed by exposure time. While short-term thermal excursions may cause minimal damage, prolonged aging, even at sub-T_g_ temperatures, can lead to significant deterioration through the cumulative effects of microcracking and chemical degradation [160]. Akay and Spratt [112] reported significant levels (30–60%) of strength loss in CFRP after extended periods of thermal aging (∼2000 h) at temperatures ranging from 0.72 to 0.86 T_g_. Based on transverse flexural tests on carbon-fiber-reinforced phenylethynyl-terminated poly(etherimide), Bullions et al. [161] noted a dependence of degradation on the level of diffusion of oxygen into the composite, resulting in the retention of transverse flexural strength at elevated temperatures being proportional to (aging time)^0.29^ using a simple power–law fit, confirming that the time of exposure was a significant driver of deterioration.

The severity of this degradation is highly dependent on the temperature level relative to the composite’s T_g_ [13,153,162]. While composites exhibit relative stability and moderate degradation below the T_g_, property loss accelerates dramatically once the exposure temperature surpasses this critical threshold. Above T_g_, the polymer matrix transitions to a rubbery [15], weakened state, affecting the integrity of the composite [153] and an increased susceptibility to premature failure modes, such as buckling [119]. This relationship is quantified by studies such as that by Jia et al. [148], who reported a nearly 70% decrease in CFRP strength at 0.95 T_g_, in contrast to a 20% loss at 0.57 T_g,_ and Bazli et al. [25], who reported deterioration of GFRP strength by only 2% at 0.75 T_g_, in contrast to a 13% deterioration at 1.5 T_g_. These findings highlight the critical role of T_g_ in defining thermal performance. Given that T_g_ can be increased through a post-process increase in temperature in a controlled manner resulting in post-cure and the consequent increase in level of polymerization and T_g_, it should be noted that specimens subjected to thermal aging can sustain a higher peak than specimens tested at ambient conditions [26]. As the levels of thermal exposure increase, in the case of a partially cured specimen, the rate of post-curing increases, and the time to reach peak flexural strength shortens. After the specimen attains the peak, a further increase in temperature or time of thermal aging accelerates mechanisms of degradation, resulting in a decline in strength [3,13,29,156]. Both flexural strength and modulus consistently decline as temperature increases [29,163], with the most severe reductions occurring near the T_g_ [13,153,162].

It should be noted that the selection of a resin with a higher T_g_ effectively delays the onset of catastrophic degradation by elevating the critical temperature threshold, as consistently observed across flexural and ILSS characteristics for both CFRP [43,103] and GFRP [164], and enhancing the quality of the fiber–matrix interphase, which is critical to decreasing the levels of thermal degradation [43,165]. To ensure the durability of the interphase and the overall composite, it was recommended to limit service temperatures to approximately 30 °F below matrix T_g_ [166].

The importance of selection of the resin system is further underscored by the distinct thermal degradation kinetics that characterize different polymer chemistries. A comparative analysis of GFRP composites with phenolic, vinyl ester, and polyester matrices reveals significant differences in their responses to thermal stress [95]. While ultimate flexural strength loss may be comparable across these systems under severe conditions, their retention of flexural modulus shows a huge difference when heated to different heat fluxes up to 100 kW/m^2^ [167]. This divergence is a direct result of their unique degradation pathways. For instance, phenolic composites exhibit superior flexural modulus retention, retaining approximately 30% of their initial modulus even after severe thermal exposure that causes complete strength loss [168,169]. This exceptional performance is attributed to the formation of a stable, carbonaceous char layer during the decomposition process. Unlike polyester resins, which degrade progressively, phenolic resins initiate decomposition later but produce a protective char that acts as a physical barrier. These variations underscore that beyond the general principle of T_g_, the specific chemical kinetics of a matrix are also crucial in determining a composite’s long-term durability under thermal stress.

As the flexural strength of a composite is a function of both the matrix and the fibers, the selection of the reinforcing fiber also plays a critical role in the extent of thermal degradation, as carbon and glass fibers possess fundamental differences in their mechanical and thermal properties that lead to distinct degradation behaviors at elevated temperatures. A comparative study by Tefera et al. [170] on CFRP and GFRP with similar volume fractions subjected to a temperature of 100 °C showed that the flexural strength of the CFRP specimen decreased from 763.63 MPa to 68.14 MPa, retaining only 9% of its original strength. In contrast, the GFRP specimen’s strength decreased from 502.76 MPa to 53.96 MPa, showing a marginally higher retention of 11%. Since the glass transition temperature was reported to be at same level as the temperature of exposure (100 °C), it can be inferred that at this temperature, the performance of both composites is primarily governed by the degradation kinetics of the polymer matrix, which is more vulnerable to heat than either fiber type. Shekarchi et al. [153], however, reported that at a higher temperature of 350 °C (where the T_g_ of this composite was reported to be 100 °C), the thermally stable carbon fibers demonstrated a superior retention percentage of 11.01%, compared to just 7.46% for glass fibers, indicating that the inherent thermal stability of the reinforcing fibers becomes the principal determinant of residual strength at elevated temperatures.

Pavan et al. [8] reported that as fiber volume fraction increases, the lower matrix content limits the formation of cracks and delamination during thermal aging, thereby enhancing flexural strength. However, this contrasts with the findings of Akay et al. [112] for ILSS, who reported that higher fiber content specimens are more prone to microcracking due to greater residual thermal stresses arising from the increased number of fiber–matrix interfaces, indicating differences accruing from choice of test method (flexure vs. ILSS).

When loads are applied longitudinally (parallel to the fibers), the stiff fibers act as the primary load-bearing constituent, with the resin’s role being an efficient stress transfer medium [92]. Because the reinforcing fibers are significantly less susceptible to thermo-oxidative degradation than the matrix, this load path offers superior thermal stability [171]. In contrast, the strength of a transversely loaded specimen is fundamentally governed by the resin matrix. The inherent lower load-carrying capacity and high thermal vulnerability of the matrix result in a significant reduction in strength as the exposure temperature increases [92]. Furthermore, transversely loaded specimens have a higher order of magnitude of exposed fiber ends, which provides preferential pathways for oxygen to enter the material and accelerate the degradation process along the fiber–matrix interface [171]. Haque et al. [171] reported that under prolonged aging at 260 °C, unidirectional CFRP laminates initially experience a strength gain due to post-curing but eventually undergo severe degradation, losing approximately 64% of the modulus at 3000 h. In contrast, when tested in the transverse direction, a 96% loss of modulus is noted within the first 1200 h. This same principle applies to GFRP, where specimens loaded longitudinally exhibit significantly higher strength retention under thermal exposure compared to those loaded transversely [92,172] and at 45° [172].

Beyond the direction of loading/testing, the macro-level arrangement of fibers, or the fiber architecture, play a decisive role in composite performance. Laminates reinforced with unidirectional, or uniformly aligned, fibers exhibit superior strength compared to those reinforced with woven fabrics [25,173], randomly oriented mats [25], or quasi-isotropic and cross-ply quasi-isotropic layups [56] across a range of elevated temperatures. This enhanced performance is attributed to the continuous and aligned nature of fibers in unidirectional laminates, which facilitates more efficient and uniform stress distribution under flexural loading. These experimental findings align with composite micromechanics principles, such as Krenchel’s strengthening factors (reinforcing efficiency), which quantify relative strength contributions as approximately 1.0 for unidirectional fibers, 0.5 for woven fabrics, and 0.38 for randomly oriented mats [174]. The practical implications are exemplified by hybrid composites, where the introduction of a single bundle of unidirectional fibers into a predominantly woven laminate increased the fiber volume fraction from 22% to 28%, resulting in a nearly 10% enhancement in strength [173].

The span-to-depth ratio (l/d) is a critical parameter that governs the dominant failure mode of a composite under flexural loading. At room temperature, specimens with lower l/d ratios typically exhibit higher flexural strength because the short, thick geometry induces a high concentration of interlaminar shear stress. This shear-dominated behavior, sometimes referred to as “arch action,” is resisted by the strong fiber–resin interfaces. However, this same characteristic becomes a critical vulnerability under thermal loading. The fiber–resin interface, which governs the shear-dominated failure mode, degrades significantly with heat, leading to a precipitous loss of interlaminar shear strength. In contrast, composites with a higher l/d ratio are more susceptible to flexure-dominated failure, where the load-carrying capacity is determined by the tensile strength of the fibers and the overall compressive strength of the composite. Since the tensile strength of the thermally stable fibers is less affected by heat, these specimens retain a greater portion of their load-carrying capacity at elevated temperatures [92].

Similarly, specimen thickness is also a critical factor for flexural performance under thermal loading, as thinner specimens allow heat to penetrate more quickly, resulting in a faster deterioration of the composite’s properties [25]. However, the effect of thickness can be altered by fiber volume fraction since fibers are far more resistant to heat than the polymer resin matrix. Additionally, specimens with lower fiber volume fractions have a higher matrix content, which increases susceptibility to cracks and delamination at elevated temperatures [8].

The accurate characterization of the effects of thermal aging on flexure necessitates a clear distinction between in situ measurement of properties at service temperature and residual properties measured after cooling to room temperature. Lv et al. [127] reported on a series of tests on CFRP exposed to levels of 150 °C for up to 1000 h, with specimens tested in situ at 150 °C exhibiting a minimum of 13% lower strength than identical specimens cooled and tested at 25 °C, along with a change in the material’s failure mode. At room temperature, the increased stability of the polymer matrix results in a brittle fracture mode characterized by tensile stress concentrations, fiber pull-out, and fracture. In contrast, when tested at the elevated temperature level, the matrix’s viscoelastic properties allow it to deform, shifting the failure mechanism to a tougher, compressive-side failure characterized by delamination and loss of cohesive strength between fibers and resin. A similar observation was also reported by El-Gamal et al. [155] for GFRP. The intricate relationship between composite processing history and subsequent mechanical performance under service conditions is elucidated by Sun et al. [124] through an investigation of the effect of varied post-cure temperatures (315 °C to 375 °C) on composite mechanical properties. The strength of the specimen when tested at room temperature was found to be higher than when tested at 400 °C.

**Figure 12:**
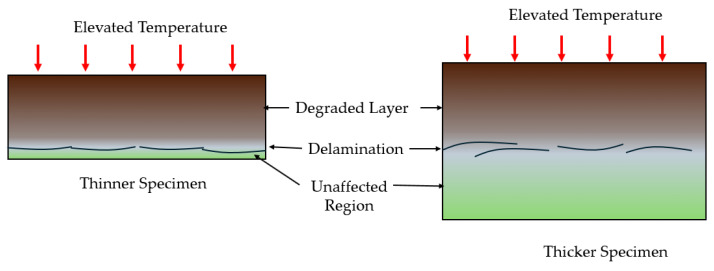
Effect of thickness of the composite.

Along with time and temperature, the role of the environment is also important. Under oxidative conditions, the formation of a thermally oxidized layer (TOL) forms a barrier to further permeation and does not result in a decrease in the flexural strength of CFRP even after the temperature crosses the glass transition [151]. However, this layer only helps to attenuate the effect of oxygen but does not prevent the composite from degradation, with degradation being accelerated at higher levels of oxygen partial pressure by promoting faster matrix oxidation [161]. It should be noted that under elevated temperature conditions, the extent of degradation is dependent on thickness, with oxygen-limited surface degradation and relatively less affected core regions. In thick laminates and wet layup systems with resin-rich regions, including at the surface and between fabric layers in a stack, oxidative degradation and matrix embrittlement are concentrated near exposed surfaces, while the interior experiences reduced oxygen availability, leading to non-uniform deterioration, as a consequence of which there is non-uniform shear performance and surface-mechanism-driven failure initiation. As both the temperature and duration of thermal aging increase, the extent of material deterioration correspondingly intensifies. During this process, laminate thickness becomes a governing parameter, influencing both the severity of degradation and the depth to which heat-induced damage penetrates the composite laminate, as thinner laminates permit more rapid heat penetration, leading to accelerated deterioration of mechanical properties [25]. When the surface of a polymer–matrix composite is subjected to intense radiant heating, a steep through-thickness temperature gradient develops due to the inherently low thermal conductivity of polymer matrices, resulting in a mismatch in thermal expansion between the highly heated surface layers and the relatively cooler underlying material, which generates significant interlaminar thermal stresses that can initiate delamination cracking [88] as shown schematically in Figure 12.

The flexural modulus of FRPs is noted to show less deterioration than strength at elevated temperatures for both CFRP [43,148,170] and GFRP [149,154,175]. Nema et al. [43] reported that the flexural modulus remained nearly constant up to 170 °C, after which it began to decrease sharply, in contrast to the flexural strength, which had already dropped by almost 50% at a temperature of 170 °C. Tefera et al. [170] observed that at 100 °C, modulus retention was still 30% for CFRP and 37% for GFRP, whereas strength retention was only 9% and 11%. This disparity reflects the distinct roles of fibers and the matrix in flexural behavior. The modulus is fiber-dominated, and since fibers remain structurally stable under heat, degradation is slow, sometimes even showing slight increases at moderate temperatures due to matrix densification or physical aging [151]. The relative stability of the modulus reflects its dependence on the intact fibers and the comparatively low longitudinal stresses they sustain [148]. Flexural strength, however, is more matrix-dependent and therefore deteriorates rapidly. As the polymer matrix softens under thermal exposure, its ability to provide lateral support and prevent fiber buckling in compression is lost. This weakened matrix support and reduced fiber–resin adhesion causes strength to drop sharply, often well before the fibers themselves are compromised [23,148,176]. At higher temperatures, where the matrix transitions into a rubbery state, fiber bending and poor load transfer further accelerate this decline [148]. This reduced load transfer due to weakened fiber–resin adhesion also contributes to a decrease in the modulus, albeit to a lesser extent than the strength degradation [177]. Transverse flexural properties, however, show a different trend. The transverse modulus suffers considerable degradation with increasing aging time due to surface damage. Since transverse flexural strength is matrix-dominated, its modulus is also strongly affected by microcracking, which reduces the effective load-bearing cross-sectional area of the composite [163].

Taken together, results from ILSS, IPSS, and flexure-based shear assessments indicate that interlaminar-dominated configurations are consistently the most sensitive to thermal exposure, particularly near and above T_g_. However, the apparent severity of degradation varies substantially with test method, specimen geometry, and thermal protocol, especially as related to the level of temperature exposure and T_g_ as summarized in Table 4.

While ILSS tests tend to indicate earlier and steeper reductions due to matrix- and interface-controlled failure, in-plane shear and flexure tests may mask degradation through stress redistribution and competing load paths. In general, ILSS tests exhibit the earliest loss of reliability as a result of matrix and interface-level degradation, while in-plane shear and flexural-based shear tests retain apparent validity at higher temperatures with stress redistribution and competing load paths. Beyond the glass transition temperature, measured shear characteristics increasingly reflect matrix flow and effects of changes in failure mode rather than intrinsic material resistance. These differences highlight that shear degradation ranking across systems and temperature regimes are inherently test-dependent and must be interpreted within the context of the activated failure mechanisms rather than the nominal strength values alone.

## 4. Effects of Non-Uniform Temperature (Cyclic and Spike) Regimes

While the effects of uniform or slowly increasing thermal exposure are fairly well characterized, actual service conditions rarely involve monotonic temperature regimes. Systems and components are more realistically subjected to repeated thermal cycles arising from diurnal fluctuations, operational variations between heating and cooling, or transient cycles, as well as short-duration high-temperature spikes accruing from localized heat sources. These non-uniform thermal histories impose fundamentally different thermomechanical demands on the composite and cause significantly different damage mechanisms and sequences of deterioration than those resulting from steady elevated temperature exposure/aging. To avoid ambiguity, it is noted that the thermal aging discussed in this section refers to time-dependent degradation under cyclic or short elevated temperature spikes below decomposition thresholds. Short-duration high-temperature excursions are treated only insofar as they influence shear response without invoking combustion-driven damage or post-fire behavior.

Cyclic high-temperature exposure produces recurrent thermally induced strains associated with the mismatch in coefficients of thermal expansion between the fiber and resin. Repeated excursions between ambient (or moderate) and elevated temperatures result in progressive matrix microcracking, interfacial debonding, and ply-level delamination (or interlayer separation). All of these show accumulation that is similar to that from fatigue loading. Thermal–mechanical interactions accelerate the degradation of matrix-dominated characteristics such as interlaminar shear strength, transverse modulus, and through-thickness toughness beyond levels that would be expected from isothermal aging even at the highest temperatures of the cycle. Further, cyclic opening and closing of microcracks results in enhanced diffusivity and permeability, which result in faster ingress of moisture and/or oxidative species into the composite, which can cause accelerated degradation.

Spikes or rapid high-temperature events generate extreme transient thermal gradients through the composite thickness. In the context of this review, thermal spikes refer to short-duration, non-combustive temperature excursions below thresholds of decomposition. This rapid rate of temperature rise produces thermal shock that can induce interlaminar stress, localized resin softening/decomposition, surface ablation, and even localized loss of fiber–matrix bond. Depending on the severity of the spike and its duration, the effects can be confined to areas close to the surface or can propagate into the thickness, resulting in the development of non-uniform damage zones and irreversible reductions in performance characteristics. Both cyclic and spike exposures represent critical, and under-researched, modes of thermal loading. Understanding the distinct mechanisms activated by these non-steady thermal regimes is essential for accurate prediction of composite response and for design. Given that the effects are emphasized in the resin and at the fiber–resin interface, effects on shear response are expected to be significant and need to be investigated further since very limited information is currently available.

The response of composite materials to thermal spiking and cycling is fundamentally controlled by two competing mechanisms of post-curing and deterioration [178]. While post-cure, or additional cross-linking of the polymer matrix, can enhance mechanical properties, this effect is often transient. As thermal exposure (i.e., the temperature of the thermal spike) increases, degradation becomes the dominant mechanism. For instance, CFRP panels exhibit remarkable resilience to temperature variations below their T_g_, with dry panels thermally spiked at 180 °C (T_g_) showing no observable decline in ILSS [179] or, as reported by Collings and Stone [180], very minimal (7%) deterioration of ILSS after cyclic exposure to 135 °C (T_g_) without showing any visible damage. However, once the thermal spike exceeds this threshold, permanent degradation occurs, as evidenced by a 25% reduction in strength after exposure to an aggressive spike of 350 °C [179]. Exposure to such aggressive conditions can cause permanent degradation, as shown by the formation of char on the specimen surface [178]. This suggests that a critical threshold of temperature and holding time exists, beyond which extensive and irreversible degradation occurs.

The rate at which a composite is cooled following a thermal spike is also a critical factor. A slower cooling rate prolongs the composite’s exposure to high temperatures, facilitating additional polymerization within the matrix and thereby enhancing fiber–matrix adhesion. This molecular restructuring results in a rapid initial increase in ILSS, as the beneficial effects of post-cure dominate over residual thermal stresses [181]. However, even with these benefits, prolonged exposure to high temperatures inevitably leads to degradation. A study by Bera et al. [182] on GFRP specimens showed about 30% deterioration in strength when subjected to a thermal spike of 100 °C, with the level of deterioration increasing significantly with a rise in temperature, driven by the formation and propagation of micro-voids and cracks, which originate from internal stresses caused by the uneven thermal expansion of the composite’s constituents. Over time, these microcracks coalesce, progressively compromising the material’s structural integrity [182]. Moreover, the significant difference in the coefficients of thermal expansion (CTEs) between the glass fibers and the epoxy matrix generates internal thermal stress, inducing strains and potentially causing localized loss of adhesion and weakening of the interface, thus leading to a decrease in ILSS [181].

The ILSS of both CFRP and GFRP becomes highly sensitive to the loading rate after thermal spiking. Thermal spikes, combined with different holding times, induce post-cure, which initially increases ILSS by enhancing the resin’s crosslink density and strengthening the fiber–matrix interphase. However, the subsequent mechanical response exhibits a complex dependence on the material’s ductility, with both CFRP and GFRP showing a rapid initial increase in ILSS up to a specific crosshead speed, which is influenced by the thermal spiking temperature and holding time for each specimen. Beyond this critical crosshead speed, the ILSS may decrease, remain plateaued, or continue to increase in CFRP, whereas the ILSS for GFRP demonstrates a continuous, albeit slower, increase in strength [178].

Based on tests conducted on carbon-fiber-reinforced cynate ester composites subjected to thermal cycling with peaks at 150 °C and 204 °C at 10 min/cycle for periods up to 5 days, Lee and Holl [183] reported that the degradation rate of IPSS on static (isothermal) exposure was nearly identical to the degradation observed under cyclic thermal exposure at the same peak temperatures as a function of exposure time. They proposed that the total effect of thermal cycling could be effectively modeled as the cumulative damage incurred during an equivalent duration of static thermal exposure at the cycle’s peak temperature, suggesting that the rate-limiting step for degradation is not the mechanical propagation of microcracks but rather the underlying chemical rate of thermo-oxidation. This indicates that, at least for the system tested, chemical breakdown of the matrix is the primary factor controlling property loss, a process whose total effect is dependent on the cumulative time at a specific temperature, regardless of whether that exposure is continuous or cyclic.

Qiu et al. [184] reported that the in-plane shear modulus of carbon fiber/epoxy laminates is highly susceptible to degradation when subjected to thermal cycling environments, with the degradation being characterized by a rapid initial decline in the modulus, followed by a stabilization or plateauing of the value after approximately 500 thermal cycles out of a total of 1000 cycles. This behavior suggests that the primary damage mechanism related to fiber–matrix debonding is due to the substantial mismatch in the CTE between the carbon fibers and the epoxy matrix, which induces shear stress at the interface during temperature fluctuations and reaches a saturation point after which further degradation due to thermal fatigue is significantly reduced.

Hough et al. [185] reported on the importance of resin selection as related to transverse flexural behavior under thermal spiking (100–200 °C) and long-term conditioning (up to 10,000 h) based on two thermoset matrix composites reinforced with carbon fibers: (i) a BMI-modified epoxy resin (Narmco Rigidite 5245C) and (ii) a high-temperature modified epoxy blend (Fibredux 927). The 927 laminate showed insignificant changes in flexural strength across the spike temperature range, whereas the 5245C laminate exhibited a modest (<5%) reduction up to 140 °C followed by a pronounced ~30% degradation between 140 °C and 160 °C.

Feng et al. [149] demonstrated that thermal cycling is considerably less detrimental to an E-glass/epoxy composite’s flexural properties than sustained isothermal exposure. When specimens were subjected to 90 days of cycling between 45 °C and 130 °C (T_g_ = 91.8 °C), with each cycle lasting up to 24 h, flexural strength decreased by only 10%, while flexural modulus increased by 22% because of ongoing post-cure during the cooler phases of the cycle. The failure mode consistently involved fiber tensile fracture at midspan, identical to that of the control specimens, indicating that the fundamental load-bearing mechanism remained constant. In contrast, under steady-state exposure at 120 °C for just 1 h, the matrix underwent complete breakdown, resulting in approximately 90% loss in both strength and modulus.

Prolonged thermal cycling has been noted to induce a progressive, cumulative degradation of flexural properties driven by the gradual accumulation of microstructural damage. Shivakumar et al. [186] reported that the flexural modulus of a carbon fiber/cyanate ester composite remained stable for up to 100 cycles, with subsequent cycling to a level of 800 cycles resulting in deterioration by 25%. This progressive damage was attributed to the combined effects of thermally induced stresses from the CTE mismatch between fiber and matrix as well as chemical degradation (oxidation) of the matrix at the peak temperature. The defects became widespread as the number of cycles increased, leading to matrix shrinkage and eventual matrix loss due to a combination of thermal stresses from the CTE mismatch between the fiber and the matrix as well as thermal oxidation/degradation of the resin in the presence of air. Although post-curing was observed (evidenced by a 60 °C increase in T_g_), this beneficial effect was insufficient to offset the accumulating microstructural damage.

**Figure 13:**
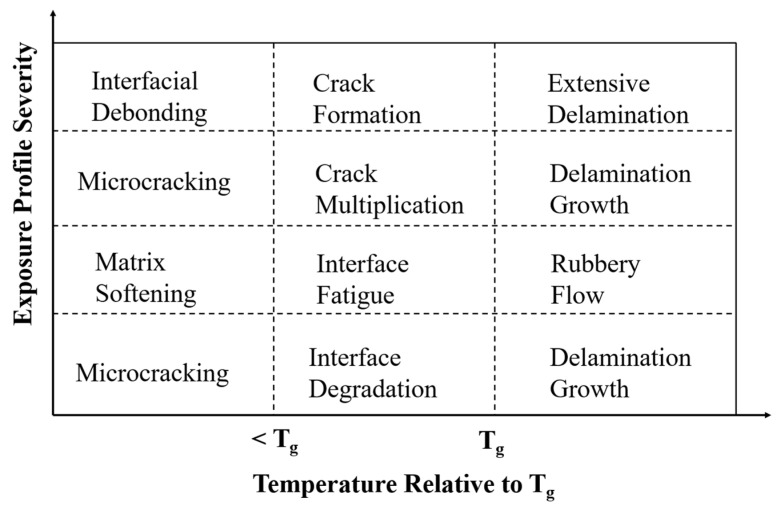
Unified thermal damage map based on exposure profile severity (severity is expressed qualitatively in terms of shear response degradation and associated transitions in failure modes. It does not include post-fire and combustion-induced damage).

Figure 13 presents a unified thermal damage map that integrates temperature regime relative to the glass transition temperature, and Table 5 indicates this with the exposure profile severity (uniform, cyclic, spike) synthesizing the dominant matrix, interphase, and delamination/separation behavior discussed earlier.

The literature on the shear response of FRP composites and effects of thermal exposure thereof reveals several unresolved contradictions and open questions that limit generalization. One recurring inconsistency concerns the reported onset temperature for critical shear degradation. While many studies identify a sharp decline in interlaminar shear strength at temperatures approaching T_g_, others report significant reductions at T/T_g_ ratios as low as 0.65–0.75, particularly in wet layup systems with resin-rich interlaminar regions. This discrepancy suggests that T_g_ alone is an insufficient predictor without explicit consideration of cure state, fiber volume fraction, and test method sensitivity. Contradictions are also evident in post-T_g_ behavior. In some studies, apparent retention, or plateauing, of shear strength above T_g_ is reported, particularly resulting from in-plane shear or flexural tests. However, detailed examination of failure modes reveals that such behavior often corresponds to matrix softening, fiber reorientation, or stress redistribution rather than true changes in load-carrying capability. Similarly, time-dependent effects remain ambiguously reported, with some studies emphasizing exposure duration as the dominant variable, while others highlight temperature as the primary driver even at short periods of exposure. These inconsistencies emphasize the need for the development of unified frameworks that explicitly distinguish reversible thermo-viscoelastic softening from irreversible chemical aging so as to reconcile test-specific sensitivities with underlying degradation mechanisms.

## 5. Analytical Models

While a large number of test methods have been developed to evaluate the shear performance of composite materials, each method carries its own set of advantages, limitations, complexities, and potential sources of inaccuracy [31]. Factors related to the machine (calibration, grips, and fixtures), specimen (dimensions, surface finish, homogeneity), and environmental factors (temperature, humidity) can compromise the repeatability and reliability of the test results. At times, these inconsistencies may lead to experimental outcomes that are not reproducible [187]. Similarly, if the material has not been tested previously, the constants required for the evaluation of stress limits may not be readily available [188]. In all these cases, analytical models can be critical in describing the behavior of materials by relating various parameters and conditions [189]. A number of analytical models have been developed to predict the thermal response of composites. Although most were originally not developed specifically for shear-related properties, such as ILSS, IPSS, or flexural behavior, some are formulated with sufficient generality to be applicable across a broad range, while others have been successfully adapted to predict, interpret, or validate shear-related experimental outcomes. Although some models may not provide absolute values for shear strength with high precision, they can serve as a valuable predictive tool, offering either a first-order estimation, a benchmark for comparison, or at the very least a prediction of trends of composite behavior under conditions of thermal aging. The major models are included in this section to provide background and a framework for the interpretation of experimental data and to guide future material design and testing strategies in environments that include thermal aging and exposure conditions. It should be noted that the focus is on thermal aging rather than the effect of exposure to fire, although there has been significant separate effort in the area of fire.

In 1981, Griffis et al. [190] developed a one-dimensional finite difference model to simulate thermal behavior and mass loss of fiber-reinforced organic composite plates under intense surface heating, such as laser irradiation, accounting for the effect of fiber ablation, matrix decomposition, and radiation and convective heat losses, demonstrating that the numerical results from their analysis aligned well with experimental data, including mass loss and thermocouple measurements from laser irradiation tests on AS/3501-6 graphite epoxy coupons. This is often considered the first model to be developed for a fiber-reinforced organic matrix composite [191]. In 1985, Chen et al. [192] extended the work done by Griffis et al. [190] by developing a finite element model to predict the failure of graphite/epoxy laminates under combined mechanical and thermal loading using nine-node Mindlin-type plate element theory and temperature-dependent material properties and strengths in conjunction with the maximum stress criterion for the prediction of failure. While the failure process was well described, the initial predictions for failure time were shorter than those given by experimental values due to the large out-of-plane deflection that violated the use of small deflection theory. In 1986, Griffis et al. [193] extended the initial model to predict the degradation in strength and structural failure of composite laminates under simultaneous exposure to intense heat and mechanical loading conditions. The model had some critical limitations in its inability to predict interlaminar failure, the assumption of linearity in the in-plane strain component, and the arbitrary extrapolation of data for fiber-dominated strength and stiffness above 480 °C, while using a threshold of 175 °C for resin-dominated characteristics such as transverse and shear properties.

Springer [194] adapted a model developed for wood during fire to provide an analytical framework for fiber-reinforced organic matrix composites exposed to elevated temperatures by correlating mass loss with the reduction in mechanical properties as

(2) $$\frac{P_{xy}}{P^{o}}=1-{\left(\frac{∆m_{xy}}{∆m_{m}}\right)}^{e}$$

where *P_xy_* and *P^o^* are the strength at time t at the specific coordinate (*x*,*y*) within the material and at room temperature, respectively. ${∆m}_{xy}$ is the mass loss at time t at the same *x*,*y* coordinate, and $∆m_{m}$ is the maximum mass loss at the end of the time period of exposure. *e* is a constant to be determined experimentally, which depends on the material and is independent of geometry and temperature but may vary with temperature if the temperature range is very wide. Failure time was derived in the form of a stress ratio, and it was noted that the specimen fails when the value of stress ratio “*R*”,

(3) $$R=\frac{\left(\frac{S_{b}}{S_{b}^{o}}\right)}{\left(\frac{M}{C\times S_{b}^{o}}\right)}$$

reaches a prescribed minimum value *R_min_*, which is generally taken to be unity. $S_{b}$ and $S_{b}^{o}$ are the maximum allowable extreme fiber stresses in bending and the extreme fiber stresses prior to exposure to elevated temperature, respectively. *M* is the maximum applied bending moment, and *C* is the section modulus. While Springer’s model laid a critical foundation for further analytical development, it had several limitations, including incomplete validation and use of a mix of composite and wood data along with the incorporation of an empirical constant “*e*”. Additionally, Springer’s analytical framework is primarily phenomenological and does not explicitly represent the underlying physical or micromechanical mechanisms governing composite degradation. The model assumes that strength degradation is entirely governed by mass loss and establishes an empirical relationship between residual strength and mass loss. However, the mass loss considered is restricted to resin volatilization, such as the loss of low-molecular-weight species (e.g., monomers and plasticizers), while neglecting other mechanisms such as moisture evaporation. Although the model acknowledges the complexity of polymer pyrolysis, it oversimplifies the process by approximating all degradation reactions through a single-step Arrhenius bulk reaction, thereby neglecting the multi-stage nature of thermal decomposition.

Mouritz and Mathys [167] focused on post-fire tensile and flexural residual properties, assuming that a fire-damaged composite can be treated as a bi-layer material consisting of a thermally degraded char layer and an underlying uncharred composite layer, such that the flexural modulus (*E_f_*) and flexural failure load (*P_f_*) can be expressed as:

(4) $$E_{f}=\left\{\frac{4{\left(d-d_{n}\right)}^{3}+4{\left(d_{n}-d_{c}\right)}^{3}}{d^{3}}+4\frac{E_{f\left(c\right)}}{E_{f\left(0\right)}}\frac{\left[d_{n}^{3}-{\left(d_{n}-d_{c}\right)}^{3}\right]}{d^{3}}\right\}{\left(EI\right)}_{f(0)}$$

where

(5) $$d_{n}=\frac{\left(E_{f\left(0\right)}d^{2}\right)-d_{c}^{2}(E_{f\left(0\right)}-E_{f\left(c\right)})}{\left(2E_{f\left(0\right)}d+2E_{f\left(c\right)}d_{c}-2E_{f\left(0\right)}d_{c}\right)}$$

and

(6) $$P_{f}=\frac{8}{L}\left\{\frac{\sigma_{f(0)}b}{3}\frac{{\left(d-d_{n}\right)}^{3}+{\left(d_{n}-d_{c}\right)}^{3}}{\left(d-d_{n}\right)}+\frac{\sigma_{f(c)}E_{f(c)}b}{3E_{f(0)}}\frac{[d_{n}^{3}-{\left(d_{n}-d_{c}\right)}^{3}}{\left(d-d_{n}\right)}\right\}$$

where $E_{f\left(c\right)}$ and $E_{f\left(0\right)}$ are the flexural modulus of the fully charred composite and uncharred composite, respectively; d, $d_{c}$, and $d_{n}$ are the composite thickness, char layer thickness, and the distance from the outer surface of the char layer to the neutral stress axis of the beam, respectively; *L* is the load span; b is the beam width; and $\sigma_{f(c)}$ and $\sigma_{f(0)}$ are the flexural strengths of charred and uncharred composite, respectively. A major limitation of this model is its core assumption that degradation is solely a function of char thickness, and thus, it does not account for delamination cracks, which are also a significant contributor to structural weakening. Furthermore, the model assumes that mechanical properties remain unaffected until the onset of charring, implying zero degradation prior to visible char formation. This assumption contradicts experimental evidence, which shows significant degradation of mechanical properties occurring well before charring, particularly due to matrix softening and interfacial deterioration.

Mahieux and Reifsnider [195] developed a Weibull-based statistical model applicable to a wide range of polymers across their entire temperature spectrum by quantitatively describing stiffness change across the transition regions when the polymer is exposed to a thermal regime and is built under the concept of bond breakage, where secondary bonds fail progressively with increasing temperature due to an increase in relaxation, leading to a change in the property of interest as

(7) $$P=\sum_{i=1}^{N}P_{i}exp\left(-{\left(\frac{T_{i}}{T_{{ref}_{i}}}\right)}^{m_{i}}\right)$$

where *P* is the material property at temperature *T*, and *N* represents the number of transitions that the material undergoes. In this study, three transitions are considered: β-transition, glass transition, and flow transition. Equation (7) is accordingly divided into three separate parts as shown in Equation (8). Since $P_{i}$ is the magnitude of the transition step or the drop in a property; *P*_1_, *P*_2_, and *P*_3_ represent the instantaneous stiffness values at the beginning of each plateau or region (e.g., beta, glass, and rubbery). $T_{i}$ is the temperature at each transition; $T_{{ref}_{i}}$ is the reference temperature at each transition; and $m_{i}$ is the Weibull modulus representing the statistics of the bond breakage so that

(8) $$P=\underset{\beta -\mathrm{transition}}{\underbrace{\left(P_{1}-P_{2}\right)exp\left(-{\left(\frac{T}{T_{1}}\right)}^{m_{1}}\right)}}+\underset{\mathrm{Glass}\;\mathrm{transition}}{\underbrace{\left(P_{2}-P_{3}\right)exp\left(-{\left(\frac{T}{T_{2}}\right)}^{m_{2}}\right)}}+\underset{\mathrm{Flow}\;\mathrm{transition}}{\underbrace{\left(P_{3}\right)exp\left(-{\left(\frac{T}{T_{3}}\right)}^{m_{3}}\right)}}$$

Correia et al. [133] calibrated this model using experimental data for GFRP composites to find the best fit for tensile, shear, and compressive strength with absolute mean percentage error (AMPE) of 4.1%, 18.0%, and 25.8%, respectively, and reported that this model provided a reasonably accurate degradation pattern of the above-mentioned mechanical properties. Despite this, it should be noted that the biggest challenges with this model are the determination of Weibull coefficients for a range of different composites since the data used were only from a single system and the fact that the model is designed to describe the instantaneous, or rate-independent, response of the material, rather than the rate or time-dependency of the property, which is critical under prolonged thermal exposure.

Gibson et al. [196] developed a semi-empirical model by integrating a comprehensive thermal analysis with classical laminate theory, avoiding the use of higher-order polynomials for property–temperature relationships, which were shown to be unreliable outside their fitted range, by Mahieux and Reifsnider [195] by using a simpler hyperbolic tangent function and proposed power–law factor ($R^{n})$ to account for the effect of residual resin content on mechanical properties such that

(9) $$P\left(T\right)=\frac{P_{U}+P_{R}}{2}-\frac{P_{U}-P_{R}}{2}tanh\left(k\left(T-T^{\prime }\right)\right)R^{n}$$

where *P(T)* is the particular property under consideration at temperature *T*, $P_{U}$ and $P_{R}$ are the unrelaxed (low temperature) and relaxed (high temperature) values of that property, $T^{\prime }$ is the glass transition temperature, *k* is the fitting parameter which signifies the extent of relaxation or the breadth of distribution, *R* represents the residual resin content and has a value between 1 (undamaged) and 0 (fully decomposed), and *n* is the sensitivity of the given mechanical property to that resin loss. In the case of tension, where reinforcement strength is only considered, “*n*” can be 0, while, if the matrix-dominated properties are only considered, “*n*” can be 1. The model showed good agreement with experimental data for compressive failure but was overly conservative in predicting tensile failure due to the model’s simplifying assumption of linear elasticity, which fails to capture the significant nonlinear behavior of woven composites in tension. In addition, the formulation is based on classical laminate theory, which assumes a plane stress state within each ply and neglects interlaminar stresses. Yu and Kodur [197] adapted the general form of the Gibson et al. [196] model and modified it by normalizing by the value of the characteristic order consideration in the unrelaxed state “*P_U_*” and fitting the equation to data of near-surface mounted (NSM) CFRP strips and rods to calculate the value of empirical constants to obtain average errors of 7% and 6.3% in tensile strength and 10% and 11.2% in elastic modulus of the strip and rod, respectively. Similarly, Hawileh et al. [198] used the general form of the Gibson et al. [196] model and used least square regression analysis to create a new set of empirical relations for their wet-layup and hybrid composite laminates by calibrating the coefficients with their own experimental data with a Net Mean Square Error (NMSE) of 0.00003 in both elastic modulus and tensile strength prediction in a CFRP laminate. Liu et al. [199] fitted the compressive stiffness and compressive strength of the individual CFRP composite rods that make up the truss cores as a function of temperature using the Gibson et al. [196] model with good agreement. Chowdhury et al. [200] calibrated the Gibson et al. [196] model for GFRP composite, with tensile strength (R^2^ = 0.9), tensile modulus (R^2^ = 0.65), and lap-splice bond shear strength tests under steady state conditions (R^2^ = 0.96) and transient conditions (R^2^ = 0.94) for up to 200 °C. They use 1 as a value of R_n_, assuming that the resin does not undergo any significant amount of degradation until 350 °C. Correia et al. [133] also calibrated this model using their experimental data to find the best fit for tensile, shear, and compressive strength of GFRP composites with absolute mean percentage errors (AMPEs) of 4.1%, 15.6%, and 23.9%, respectively. Wang et al. [201] reported that when applied to CFRP plates in tension, the model showed a very good fit up to about 400 °C (R^2^ = 0.93) but was limited to that temperature range only.

Bisby [202] adapted a sigmoidal function to model the thermal degradation of mechanical properties (elastic modulus and tensile strength) using a single, consistent equation across material types:

(10) $$\frac{P}{P_{o}}=\left(\frac{1-a}{2}\right)\tanh\left(-b(T-c\right))+\left(\frac{1+a}{2}\right)$$

where *P* is the mechanical property (strength, stiffness or bond) at temperature T; $P_{o}$ is the room temperature value of that mechanical property; *a* is a constant representative of the residual value for mechanical property; and *b* and *c* are the empirical constants that describe the severity and central temperature of degradation with temperature, respectively, and can be derived using the least squares regression analysis. Hawileh et al. [198] reported that Bisby’s model [202] failed to accurately capture the degradation behavior of their laminates, attributing the discrepancy to variations in FRP composition and the manufacturing technique. Similarly, Wang et al. [201] also reported significant deviation in model predictions from test data for pultruded CFRP plates in tension. The Bisby model is semi-empirical in nature and employs a sigmoidal function to describe strength degradation. While effective for curve fitting, the model does not incorporate key physical parameters such as *T*_g_, viscoelastic behavior, or polymer chain scission mechanisms. As a result, it lacks a mechanistic interpretation of the degradation process.

Liu et al. [203] assumed that the shear modulus (*G*) varies with temperature in the same manner as the elastic modulus (*E*) based on the general thermomechanical behavior of polymers and polymer matrix composites, where both moduli degrade significantly as temperature approaches the T_g_, such that local shear modulus, due to a non-uniform linear temperature distribution through the thickness (*h*) of the beam (due to one-sided heat flux), is given by the equation

(11) $$G\left(y\right)=G_{o}\left[1-a_{1}\frac{Q}{K∆T_{g}}\left(-y+c\right)+a_{2}{\left(\frac{Q}{K∆T_{g}}\right)}^{2}*{\left(-y+c\right)}^{2}-a_{3}{\left(\frac{Q}{K∆T_{g}}\right)}^{3}*{\left(-y+c\right)}^{3}\right]$$

where $G_{o}$ is the shear modulus at room temperature; *Q* is heat flux; *K* is the thermal conductivity; $a_{1},\;a_{2}\;\mathrm{and}\;a_{3}$ are the fitting constants; $∆T_{g}$ is the temperature differential $\left(T_{g}-T_{0}\right)$; ∆T is the temperature rise above room temperature $\left(T-T_{0}\right)$; $T_{0}$ is the room temperature; and *c* = *K*/*H* + *h*/2, where *H* is the surface conductivity from the composite surface to the air. This model assumes a linear, steady-state temperature distribution through the composite thickness rather than the reality of the temperature evolving dynamically with time, resulting in the development of non-linear thermal gradients. The steady-state assumption, therefore, limits the model’s applicability.

Bai et al. [204] developed a model for predicting the temperature-dependent mechanical properties (modulus of elasticity and shear modulus) of pultruded GFRP composites based on kinetic theory, hypothesizing that a composite can be treated as a mixture of different material states at any given temperature, with mechanical properties (modulus or viscosity) being defined in four distinct states: glassy by *P_g_*, leathery by *P_l_*, rubbery by *P_r_*, and decomposed by *P_d_*, such that the overall property is given as

(12) $$P_{m}=P_{g}\left(1-\alpha_{g}\right)+P_{l}\left(1-\alpha_{r}\right)\alpha_{g}+P_{r}\left(1-\alpha_{d}\right)\alpha_{g}\alpha_{r}+P_{d}\alpha_{g}\alpha_{r}\alpha_{d}$$

where $\alpha_{g},\;\alpha_{r},\;{\;\mathrm{and}\;\alpha }_{d}$ are conversion degree of glass transition, leathery-to-rubbery transition, and rubbery-to-decomposed transition, respectively, which were derived from kinetic theory using an Arrhenius-type equation given by

(13) $$\frac{d\alpha_{s}}{dT}=\frac{A_{s}}{\beta }exp\left(\frac{-E_{A,s}}{RT}\right){\left(1-\alpha_{s}\right)}^{n}$$

where $\alpha_{s}$ is the degree of conversion where the subscript “*s*” denotes different states (glass transition, leathery-to-rubbery transition, and rubbery-to-decomposed), *T* is the temperature, *A_s_* and *E_A,s_* are the pre-exponential factor and activation energy for those transitions, β is the constant heating rate, *R* is the universal gas constant (8.314 J/mol K), and *n* is the reaction order, which was assumed to be 1. Correia et al. [133] calibrated this model using experimental data for GFRP composites and found that the model provided reasonably accurate predictions for tensile strength (average mean percentage error (AMPE) = 6.5 %), while its accuracy was considerably lower for shear (AMPE = 35.9%) and compressive strength (AMPE = 68.6%). While the model has a strong phenomenological basis, the use of experimental data to determine kinetic parameters, such as activation energy and the pre-exponential factor, and the limitation of 250 °C for predictions are major drawbacks.

Based on tensile tests of CFRP, pultruded plates showed two large reductions: one between 20 °C and 150 °C due to softening and gasification of the epoxy resin matrix and another from 450 °C to 706 °C due to oxidation of carbon fiber and complete removal of the epoxy matrix due to burning. Wang et al. [201] drew inspiration from models for stainless steel and cold-formed steel materials to propose a stress–temperature model with coefficients calibrated for three different temperature ranges from room temperature up to about 700 °C, as

(14) $$\frac{P\left(T\right)}{P_{u,normal}}=\left\{\begin{array}{c} 1-\left(\frac{{\left(T-22\right)}^{0.9}}{200}\right)\;\;\;\;\;\;\;\;\;\;\;\;\;\;\;\;\;\;\;\;\;\;\;\;22\leq T<150\;\;\\ 0.59-\left(\frac{{\left(T-150\right)}^{0.7}}{490}\right)\;\;\;\;\;\;\;\;\;\;\;\;\;\;\;150\leq T<420\\ 0.48-\left(\frac{{\left(T-420\right)}^{1.8}}{76000}\right)\;\;\;\;\;\;\;\;\;\;\;\;\;\;\;420\leq T<706 \end{array}\right.\;$$

where *P*(*T*) is the ultimate stress at temperature *T* (°C), and $P_{u,normal}$ is the ultimate stress at room temperature. Correia et al. [133] calibrated this model using experimental data of GFRP composites and reported absolute mean percentage errors (AMPEs) of 3.9%, 19.5%, and 18.1% for tensile, shear, and compressive strength, respectively. While the approach may provide reasonable predictions within calibrated ranges, it does not incorporate or explain the underlying physical mechanisms governing material degradation, limiting its generalization beyond the specific set of tested conditions.

Correia et al. [133] further developed a model for the accurate prediction of degradation under specific loading conditions of tensile, compressive and shear strength of GFRP composites such that

(15) $$P=\left(1-e^{{Be}^{C*T}}\right)\times \left(P_{u}-P_{r}\right)+P_{r}$$

where *B* and *C* are the shape and scale parameters of the Gompertz distribution, respectively, and can be obtained through fitting the data; *P_u_* and *P_r_* are the mechanical properties of that specimen at the unexposed condition and at its decomposed state, respectively; and *T* is the temperature. A comparison of the proposed Gompertz-based model against existing models by Mahieux et al. [195], Gibson et al. [196], Wang et al. [201] and Bai and Keller [204] showed the model to be most suited for describing the degradation of shear (AMPE = 14.2%) and compressive (AMPE = 20.1%) strengths, but having less accuracy for tensile strength (AMPE = 7.8%), limiting the model’s predictability in fiber-dominated failure mechanisms.

Guo et al. [205] developed a temperature-dependent modulus model aiming to predict both the dynamic storage modulus and static flexural modulus for a GFRP composite dependent on a single parameter (*k*), which is the intrinsic growth rate of the number of rubbery-state molecules per unit temperature.

(16) $$E\left(T\right)=\frac{E_{g}+E_{r}}{2}-\frac{E_{g}-E_{r}}{2}tanh\left[\frac{k}{2}(T-T_{g})\right]$$

where *E*(*T*) is the flexural modulus or dynamic storage modulus at temperature *T*, $E_{g}$ is the modulus of the sample in the glassy state, $E_{r}$ is the modulus of the sample in the rubbery state, and T_g_ is the glass transition temperature.

The model can be mathematically transformed into the functional form of Gibson et al.’s [196] model when resin decomposition is neglected, emphasizing formulation of the clear physical interpretation linked to molecular transitions; in contrast to its use in Gibson’s model, “*k*” is described more generally as a material constant reflecting the extent of relaxation. Furthermore, Guo et al. [205] showed that the Arrhenius-type equation proposed by Bai et al. [204] incorporates three independent parameters and does not yield an analytical solution for the degree of glass transition, whereas their proposed model required only one parameter and offered a concise, convenient analytical solution. It should be noted that the applicability of this model is limited to a temperature range below the onset of the material’s degradation, such that it describes only the thermomechanical softening that occurs during the glass transition temperature, completely avoiding chemical degradation, or mass loss, which occurs at much higher temperatures.

Feng et al. [149] recognized that existing models for FRP degradation at high temperatures are often complex, requiring extensive experimental data for curve fitting, and can be confusing in design application by structural engineers, and they proposed a simplified design method that focused on providing a conservative lower envelope of experimental data, making it safer for design applications. While useful for defining safety limits, the approach is not rooted in material physics and does not explain the mechanisms governing strength degradation. The model was validated by normalizing a wide range of experimental data for flexural, tensile, shear, and compressive strength from previous research [130,200,206,207] by the corresponding initial values at room temperature and showed that their piecewise model consistently provided a lower envelope of these data points, thus confirming its conservative nature. This model assumes that the degradation of property *P*(*T*) at temperature *T* follows a temperature-dependent behavior and can be described using a piecewise linear function. Additionally, this model incorporates the temperature threshold where T_s_ is the temperature at the start of storage modulus degradation (if lower than room temperature, then it is set to room temperature), T_g_ corresponds to the peak damping parameter, and T_r_ is the start of the residual plateau in the DMA curve for the storage modulus, such that

(17) $$P\left(T\right)=\left\{\begin{array}{c} P_{i}\\ \frac{P_{r}-P_{i}}{T_{g}-T_{s}}\;\left(T-T_{s}\right)+P_{i}\\ P_{r} \end{array}\right.\;\;\;\;\begin{array}{c} \;\;\;\;\;\;\;\;\;\;\;T\leq T_{s}\;\;\;\;\;\;\;\;\;\\ {\;\;\;\;\;\;\;\;\;\;\;\;\;\;T}_{s}<T<T_{g}\;\\ \;\;\;\;\;\;\;\;\;\;\;\;\;\;\;T_{g}\leq T\leq \;T_{r}\;\; \end{array}$$

where T_s_, T_g_, and T_r_ are all determined using dynamic mechanical analysis (DMA). The method assumes that a mechanical property maintains its initial value, *P_i_*, from −40 °C, and T_s_ decreases linearly above T_g_ until it reaches the residual value *P_r_* at T_g_. After T_g_, the property remains constant at *P_r_* until T_r_.

Feng et al. [208] also recognized that most models were designed to describe property degradation as a function of temperature without incorporation of frequency effects, which are particularly relevant in civil engineering structures subjected to dynamic loading through the introduction of a parameter “*m*” to control the asymmetry of the glass transition region such that the storage moduli is given by

(18) $$E\left(T\right)=E_{g}-\frac{E_{g}-E_{r}}{{\left[\left(2^{m}-1\right)e^{-mk\left(T-T_{mg}\right)}+1\right]}^{\frac{1}{m}}}$$

where *E* is the instantaneous storage modulus; *E_g_* and *E_r_* are the storage moduli of the sample in the glass state and rubbery state, respectively; *T* is the temperature; *T_mg_* is the temperature determined from the storage modulus at the half-height of the step change; and k is the intrinsic growth rate per unit temperature of the number of rubber-state molecules. While this was an improvement on the previous model, the dependence on experimentally determined parameters, although linked to physical interpretations, as well as the inability to predict static strength, stand out as limitations. Additionally, the model does not account for the β-transition. Specifically, the model assumes that the loss modulus (*E*″) in the glassy state is nearly zero, whereas experimental observations show that *E*″ remains non-zero due to localized side-group motion associated with the β-transition. Furthermore, the model does not address char formation or complete composite decomposition, as mass loss is not considered.

Bazli et al. [25] more recently developed a probabilistic model by systematically investigating the effects of multiple variables, including fiber orientation, laminate thickness, and exposure time, using ANOVA and a linear Bayesian regression method,

(19) $$R\left(\%\right)=a\left(\frac{1}{T^{3}}\right)+b\left(\frac{1}{{\left(Log\left(\frac{t_{1}}{6}\right)\right)}^{0.5}}\right)-c\left(\frac{1}{{\left(Log\left(t_{2}\right)\right)}^{0.5}}\right)+d$$

where *R* (%) is the retention percentage of the mechanical property; *T* is the temperature in degree Kelvin; $t_{1}$ (min) is the exposure time; $t_{2}$ (mm) is the thickness; and *a*, *b*, *c*, and *d* are the constants based on the type of laminates. While extremely well stated, the model is hindered by its limitation of a threshold of 200 °C as the upper limit, and it does not provide insight into the physical or micromechanical mechanisms responsible for degradation, thereby limiting its interpretability.

Zhou and Zhang [35] proposed a model for tensile strength of CFRP tendons to better reflect the underlying thermophysical behavior of the composites by incorporating key thermophysical properties like fiber content (*V_f_*), glass transition, and decomposition temperatures, focusing on the comprehensive effects of resin softening and decomposition as

(20) $$\frac{P(T)}{P_{o}}=\left(1-V_{f}\right)exp\left[-k_{1}{\left(\frac{T-T_{o}}{T_{g}}\right)}^{3}\right]+V_{f}\exp\left[-k_{2}{\left(\frac{T-T_{o}}{T_{d}}\right)}^{3}\right]$$

where *P*(*T*) is the mechanical property of the material at temperature (*T*); $P_{o}$ is the mechanical property of the material at ambient temperature; $T_{o}$, $T_{g}$, and $T_{d}$ are the ambient temperature, glass transition temperature, and decomposition temperature; and $k_{1}\;\mathrm{and}\;k_{2}$ are the fitted constants from experimental data. The limitation is that the model is based on the application of the rule of mixture through which the instantaneous composite strength is expressed as the sum of the remaining matrix strength and remaining fiber strength. This effectively suggests that even when the matrix fully degrades, since the fiber strength remains largely unaffected, the composite has a much higher strength, ignoring the reality of interphase degradation and loss in stress transferability.

Sengodan et al. [209] developed a finite-element framework that uses a set of phenomenological equations incorporating a modified Zhurkov’s kinetic approach to predict several matrix-dominated strength properties, including in-plane shear strength and interlaminar shear strength. Malmorad et al. [210] developed a model to calculate the flexural strength of high-capacity composite conductors used in power transmission lines at elevated temperatures using simple DMA data and a single quasi-static flexural experiment at room temperature, with the flexural strength being expressed as,

(21) $$\frac{\sigma_{f}(T)}{\sigma_{f0}}=\frac{\alpha E_{C0}\frac{E^{\prime }(T)}{E_{0}^{\prime }}}{\alpha E_{C0}+k{\left(\frac{L}{h}\right)}^{2}G_{C0}\left[1-\frac{E^{\prime }(T)}{E_{0}^{\prime }}\right]}$$

where $\sigma_{f}(T)$ is the flexural strength at temperature *T*; $\sigma_{f0}$ is the flexural strength at room temperature; α is the loading mode parameter; *k* is the shear correction factor; *L* and *h* are the span length and height of the composite beam, respectively; $E_{C0}$ and $G_{C0}$ are the composite axial young modulus and in-plane shear modulus at room temperature; and $E_{0}^{\prime }$ and $E^{\prime }(T)$ are the storage modulus at room temperature and at that temperature, respectively. Although predictions are in good agreement with the experimental data over a range of temperatures, including the glassy, transition, and rubbery stages, the model’s applicability is limited to FRPs with a high fiber volume fraction $\left(≅70\%\right)$. Furthermore, the model relies on a temperature shift factor to correlate DMA and static test data, which requires at least one elevated-temperature test for its determination, partially undermining the model’s claim of only needing room temperature data. The model also defines failure at the onset of nonlinearity in the stress–strain curve, resulting in a conservative estimate that is significantly below the material’s ultimate capacity.

Recognizing that existing models did not account for the linear decrease in flexural strength and ILSS with an increase in temperature, Nema et al. [43] developed an analytical model to predict both by using only a few parameters obtained from standard room temperature and DMA tests, such that

(22) $$\sigma \left(T\right)=\sigma_{0}\left(1-\alpha \frac{T-T_{0}}{T_{g}}\right)$$

where $\sigma \left(T\right)$ is the instantaneous temperature-dependent strength (flexural and ILSS), $\sigma_{0}$ is the corresponding strength at room temperature, α is the reduction factor, *T* is the instantaneous temperature, $T_{0}$ is the room temperature, and $T_{g}$ is the glass transition temperature. The reduction factor, α, is given by:

(23) $$\alpha =\frac{E_{g}^{\prime }-E_{r}^{\prime }}{E_{g}^{\prime }}$$

where $E_{g}^{\prime }$ and $E_{r}^{\prime }$ are the storage modulus values determined through DMA tests at the glassy and rubbery states, respectively. This model was also successfully validated with flexural strength data from Jia et al. [148]. The assumption of linearity does somewhat restrict applicability in the event of a more rapid decrease, and in addition, the model cannot be used to predict the modulus.

The general overview of analytical models for predicting composite properties in thermal environments, described briefly herein, shows that no single approach is universally optimal, with each offering distinct strengths and limitations. The literature also makes it clear that fully empirical equations, while sometimes convenient, have narrow applicability. Future work should therefore prioritize the development of models grounded in physical principles or, at a minimum, robust semi-empirical or phenomenological frameworks with broad applicability. Models such as those by Gibson et al. [196], Bai and Keller [204], Feng et al. [208] and Correia et al. [133] are excellent examples, as their broad applicability and physical basis have allowed for their successful adaptation to various scenarios despite specific limitations. Another useful approach is in the development of models that can accurately define the lower-bound envelope of strength degradation, such as the one from Feng et al. [149], which offers engineers a conservative tool for structural design, although with potentially higher costs.

Although a range of analytical and semi-empirical models have been proposed to describe thermal degradation of composite characteristics, significant mechanistic gaps remain when applied to a shear-dominated response. Arrhenius-based formulations effectively capture temperature–time equivalents for chemical aging but neglect stress redistribution, interface degradation, and geometry-dependent effects. Time–temperature superposition approaches are similarly limited, particularly near T_g_, where irreversible damage mechanisms violate the assumptions of linear viscoelasticity. Viscoelastic and rheological models capture matrix softening but fail to account for oxidation, microcrack coalescence, and thickness-dependent oxygen diffusion. As a result, existing models are unable to distinguish between reversible thermo-viscoelastic effects and irreversible chemical degradation. Further, they are unable to reliably predict transitions in failure modes. These limitations emphasize the need for physically informed, multi-mechanism frameworks that explicitly incorporate matrix chemistry, interphase changes, and non-uniform thermal fields.

## 6. Summary and Conclusions

### 6.1. Key Findings and Summary

From a design and practice perspective, the findings synthesized in this review reinforce the conservative temperature limits traditionally adopted for structural FRP systems. While shear response near the glass transition temperature provides valuable mechanistic insight into degradation pathways and transitions in failure modes, such conditions are not representative of intended service environments. For composites fabricated using wet lay-up and for resin-rich systems in particular, early degradation of interlaminar and interfacial shear transfer underscore the importance of maintaining service temperatures well below T_g_ and accounting for time-dependent effects in the assessment of durability. The results further emphasize that apparent strength retention observed in certain shear tests at elevated temperatures mainly reflects stress redistribution rather than true material capacity, underlining the need for cautious interpretation when extrapolating laboratory data to field applications. A critical review of the literature shows that the thermal response of FRP composites in shear is fundamentally governed by the temperature-dependent response of the polymer and the integrity of the fiber–matrix interphase. The resin-dominated response includes the critical aspects of inter- and intra-laminar responses, which are exacerbated in resin-rich zones such as between layers of fabric in wet layup-based composites and composites with lower fiber volume fraction. The dominance arises because the shear characteristics, as seen through ILSS, IPSS, or flexure, rely heavily on the resin to transfer load. Thus, shear-related degradation reflects not only on matrix softening but also the evolution of microstructural damage at the interphase and in the bulk matrix, making shear a sensitive diagnostic indicator of thermal aging [211]. Three overarching findings are noted consistently across studies.

- Temperature relative to T_g_ governs the progression of damage mechanisms. Below T_g_, the composite experiences physical ageing and stiffening, while temperatures approaching T_g_ induce matrix softening, a reduction in shear transfer capacity, and early interfacial debonding and inter-/intra-laminar separation. As the temperature reaches and exceeds T_g_, the resin enters a rubbery state, which reduces the effective shear modulus, redistributes internal stress, and increases the propensity for catastrophic delamination and separation of layers. As the temperature approaches T_d_, chain scission, thermo-oxidative degradation, and breakdown of crosslinks in the polymer network dominate, resulting in irreversible degradation.
- Details of thermal exposure profiles have a distinct impact on both the rate and spatial distribution of damage. While isothermal aging results in surface-level oxidation and slow mass loss, cyclic and spiked exposures activate fundamentally different mechanisms. Cyclic temperature regimes introduce repeated thermal expansion and contraction, resulting in fatigue-like interfacial stresses and microcracking at rates far exceeding those from steady heating. Spike, or flash, exposures result in steep thermal gradients, which lead to rapid interphase failure, initiation of localized resin charring/vitrification, and steep drops in stiffness even at temperature levels well below T_d_.
- Retention of mechanical properties is strongly dependent on constituents and laminate design. Unidirectional composites and loading in the fiber direction result in higher residual performance due to fiber-dominant modes, whereas multidirectional and transverse configurations are more sensitive to thermal degradation. Aspects such as fiber volume fraction, type of fiber, and resin type have a critical influence, especially at levels close to and above T_g_ and especially T_d_. The presence of multiple interfaces between layers of reinforcement introduces mismatch zones, especially with changing fiber orientations, resulting in interlaminar and intralaminar shears that are extremely sensitive to thermal loading.

Additionally, oxidative environments and faster heating rates magnify degradation kinetics. Oxygen availability shifts degradation from a primary physical transformation to a chemically reactive process that includes progressive embrittlement, which significantly reduces ILSS and IPSS relative to inert environments. Overall, the literature indicates that exposure to elevated temperatures results in a degradation of shear-associated properties through a complex interplay of thermally induced matrix softening, interphase weakening due to thermal expansion mismatch, and residual stress relaxation, as well as thermal deterioration of the interphasial region itself, oxidation, and thermomechanical fatigue, all of which are exacerbated as profiles deviate from steady state. The level of interaction of phenomena explains why shear characteristics often show greater and earlier degradation and at lower temperatures than the corresponding tensile and compressive properties.

### 6.2. Contradictions and Unresolved Issues

While there is broad agreement on general trends, several key contradictions and ambiguities remain unresolved.

- Uncertain definition of the “vicinity of T_g_”.
  The term vicinity has been defined using temperature effects ranging from 16–22 °C [166,212,213] and even remaining ambiguous. The inconsistency arises because the concept of T_g_ is itself not an absolute since it is a range of response and can be defined via onset, peak, or tan (*δ*) criteria, each representing a different physical transition through DMA, and is even further different when determined using differential scanning calorimetry (DSC) or thermogravimetric analysis (TGA) methods. Moreover, the degree of cure, post-cure behavior, damage, moisture uptake (or loss), and matrix chemistry can shift T_g_ dynamically during, and as a result of, thermal exposure, making it difficult to generalize mechanical response in the T_g_ region without addressing these subtleties.
- Duality and competition between post-cure and degradation
  When exposed to thermal regimes, some composites exhibit an initial strengthening effect as additional crosslinking occurs, while others degrade. Interpretations of the response suggest that the contradiction may stem from differences in the initial extent of cure, rate of stress relaxation in the matrix, whether oxygen is present, and the extent of its diffusion into the composite. Some systems may appear to gain in performance only because post-cure offsets early chain scission, masking the initiation of deterioration.
- Inconsistent effects of fiber volume fraction
  Studies show conflicting data on whether an increase or decrease in fiber volume fraction improves ILSS and flexural strength [8,112], likely due to the different governing failure mechanisms. Flexural failure is often initiated by compression or tension (fiber-dominated characteristics), whereas ILSS is largely matrix-dominated. However, at elevated temperatures, delamination/layer separation dominates across all modes.
- Contradicting findings regarding bar diameter
  Some reports cite higher residual flexural strength in large-diameter bars [15], whereas others report the opposite [155]. This discrepancy could be related to differences in bar surface condition, resin rich surfaces and even test setup.
- Unresolved origins of dual -peak IPSS response in CFRP
  A distinct dual-peak load-displacement profile has been observed in some CFRP [129] systems but not in GFRP, raising questions about whether this is a fundamental material response, a reporting gap, or even a figment of test setup.
- Persistent nonlinearity in IPSS
  The dominant contributor to nonlinearity fiber rotation, matrix shear deformation and/or interphase softening and yielding remain unclear and insufficiently studied. Of special incongruity is the presence of nonlinearity even in ambient cured materials, where one would expect greater initial linearity with increasing progression of degree of polymerization and cure.
- Conflicting assessment of the severity of thermal cycling
  Some studies conclude that cycling loading is as damaging as static exposure [183], while others report that it is less damaging and could even be initially beneficial due to incremental post-cure [149,181]. The lack of consensus regarding temperature amplitude, frequency and dwell time remains a major unresolved issue.

These issues, among others, highlight the challenges of generalizing thermal responses across different composite systems and make the development of comprehensive analytical models extremely difficult while also emphasizing the critical need for rigorously controlled and well-designed experiments that take into account the inherent variability at the materials level, especially in non-autoclave cured and ambient/moderate temperature cure-based manual processes.

### 6.3. Critical Areas for Future Study

Several key gaps were identified that require targeted investigations to advance the understanding of shear-dominated FRP behavior under elevated temperature regimes. These include:

- Expanded investigation of shear-dominated properties:
  ILSS, IPSS and flexural response under elevated temperatures remain significantly understudied relative to tensile and compressive properties, especially as related to composites fabricated using non-autoclave cure process. More comprehensive datasets are needed to characterize thermal response across differing resin chemistries, fiber types, and layup architectures.
- Systematic study of thermal cycling and spiking entirely above room temperature:
  Most available studies on thermal cycling or spike/flash loading involve cryogenic (or near-cryogenic)-to-elevated temperature ranges, leaving a critical gap in the understanding of response solely above ambient temperatures, which may be more relevant to use in industrial, offshore, naval/marine, and infrastructure applications. The effects of extended periods of expected service life and the consequent increase in the number of cycles and spike events also need to be investigated.
- Mechanistic understanding of matrix-dominated transitions near T_g_:
  Research is needed to establish clear criteria for T_g_ vicinity, quantify and elucidate the competition between thermal exposure-induced post-cure and degradation, and generalize failure mode transitions under different heating rates and exposure profiles. In addition, more detailed microstructural investigations including mechanistic modeling are needed to quantify the effects of phenomena such as thermal softening, oxidation, microcracking and microcrack coalescence, and interphase debonding, as well as their interactions.
- Integration of oxidative and environmental effects:
  Future investigations need to elucidate the roles of oxygen availability and diffusion, humidity, and thermal regime (including heating rate) and the complex interactions between these factors to provide comprehensive understanding of deteriorative mechanism accruing from these aspects.
- Clarification of emergent non-linear and dual-peak responses:
  Detailed studies on fiber reorientation, interphase softening and yielding, and matrix flow under shear, especially as temperatures approach and exceed T_g_, are necessary to explain non-linearities and dual-peak behaviors as well as differences based on fiber type, which effectively are derived based on the differences between fiber types and the anisotropy of some fibers (e.g., Carbon and Aramid).
- Development of standardized test methods and baselines:
  There exist a large number of tests, each of which introduces load in a different way and could result in the effects of thermal aging activating different mechanisms and consequently providing variations in response to the same material and loading regime based on the intricacies of the test method. In addition, mechanisms and shear characteristics are influenced by aspects such as fiber volume fraction, minor changes in resin chemistry, test specimen geometry, layup in terms of thickness and orientation, post-cure, and rate of loading, as well as heating, making it difficult, at times, to compare results. Standardization in test methods and the development of validated, detailed databases that comprehensively assess variation in test variables are essential.
- Development of physically informed analytical models:
  Existing models remain largely empirical and lack the ability to integrate temperature-dependent matrix transitions, interphase degradation, and non-uniform exposure histories. Mechanism-based, multiscale, physics-based models that incorporate both chemical and mechanical degradation processes are essential for predictive design.

### 6.4. Conclusions

This comprehensive review synthesizes a broad body of research on the thermal behavior of FRP composites, providing an understanding of how uniform and non-uniform temperature exposures affect shear-dominated properties. The core finding is the dominant role of the glass transition temperature (T_g_) in the rapid loss of stiffness and strength in composite materials as the exposure temperature approaches or exceeds this threshold. The deterioration rate is noted to be a synergistic function of both time and temperature and is further influenced by factors such as the type of matrix and fibers, fiber volume fraction, and the degree and history of the post-cure process. It is noted that the shear modulus shows less degradation than strength due to its direct dependency on the fibers, which are more thermally stable than the matrix. Shear response is often the earliest indicator of impending failure or critical deterioration in the integrity of a composite. Yet, its understanding is significantly less than that of fiber-dominated characteristics such as tension and compression. Significant research gaps persist, particularly in the determination of mechanisms, elevated temperature thermal cycling and thermal spike, hybrid reinforcement systems, and the absence of standardized in situ testing protocols. Similarly, most of the currently available analytical models are largely empirical and lack broad applicability in the diverse thermal environments that need to be assessed, underscoring the necessity for a set of phenomenological or physics-based models that are validated across a wider range of materials and exposure conditions. Collectively, the lacunae represent significant opportunities to strengthen reliability, safety, and predictive understanding of the shear response of FRP composites in elevated temperature service conditions.

## Figures and Tables

**Table 1:** Typical coefficients of thermal expansion.

	Coefficient of Thermal Expansion (× 10^−6^/°C)	
Material	Longitudinal Direction	Transverse Direction
Carbon Fiber (T300)	−0.54 [36]	10.08 [36]
Glass Fiber (E-glass)	5.3 [37]	
Resin (5208 Epoxy)	43.92 [36]	
CFRP (Unidirectional Graphite/Epoxy)	−1.0764 to −0.0018 (Vf = 0.48 to 0.68) [36]	25.25 to 47.41 (Vf = 0.48 to 0.68) [36]
GFRP (Unidirectional)	6 to 10 (Vf = 0.5 to 0.7) [18]	19 to 23 (Vf = 0.5 to 0.7) [18]

**Table 2:** Comparison of shear-related test methods under thermal aging.

Test Method	Dominant Load Path/Failure Mode	Sensitivity to Thermal Aging	Key Mechanism
Interlaminar Shear	Matrix and interface dominated, interlaminar focus	High	Matrix softening and microcracking, fiber–matrix debonding, post-T_g_ chain scission
In-Plane Shear	Combined matrix shear and fiber-dominated shear transfer	Moderate	Matrix yielding and softening, reduced shear stiffness, and increasing interfacial slip
Flexural	Coupled bending and shear, competing effects	Mixed	Matrix compression softening, creep, fiber micro-buckling, and interlaminar shear degradation

**Table 3:** Principal limitations of standardized test methods for shear characterization under elevated temperature exposure.

Test Method	Key Assumption	Breakdown at Elevated Temperature	Risk to Interpretation
ILSS	Arch action, intact interface	Matrix softening, delamination	Degradation levels can be overestimated
Iosipescu	Uniform shear zone	Notch crushing, resin flow	Invalid results for shear
In-Plane Shear (±45 IPSS)	Linear elastic response	Fiber rotation, nonlinearity	Erroneous apparent strength retention
Flexure	Stable shear-bending ratio	Mode changes	Mechanism ambiguity

**Table 4:** Relative severity of shear degradation in FRP composites as a function of temperature regime and test method.

Temperature Regime	ILSS Sensitivity	IPSS Sensitivity	Flexural-Shear Sensitivity	Dominant Mechanism
T/T_g_ < 0.7	Low	Low	Low	Physical aging, post-cure
0.7 ≤ T/T_g_ < 0.85	Moderate	Low–moderate	Moderate	Matrix softening, interfacial change and slip
T ≈ T_g_	High	Moderate	Moderate	Matrix yielding, delamination
T > T_g_ (below T_d_)	Critical	Apparent (can be misleading)	Variable	Matrix flow, stress redistribution

**Table 5:** Thermal damage effects based on thermal loading type and temperature regime.

Temperature Regime	Constant Uniform Temperature	Monotonic Increasing Temperature	Cyclic Thermal Loading	Spike/Flash Thermal Loading
Below T_g_	Oxidation	Gradual matrix softening	Progressive microcracking and interfacial damage	-
Near T_g_	Microcracking Interfacial debonding	Phase transition to rubbery state	Crack growth with cycles Oxygen ingress caused cracking	Sharp thermal gradients-based surface softening
Above T_g_ but below T_d_	Delamination	Delamination	Rapid resin softening and interfacial debonding	Localized resin decomposition Fiber oxidation
Approaching T_d_	Chain scission Significant matrix loss	Significant matrix loss	Localized resin decomposition	Surface softening Shear collapse

## Data Availability

No new data were created or analyzed in this study.
